# Circulating immune cell phenotypes are associated with age, sex, CMV, and smoking status in the Framingham Heart Study offspring participants

**DOI:** 10.18632/aging.204686

**Published:** 2023-04-27

**Authors:** Yuan Fang, Margaret F. Doyle, Jiachen Chen, Jesse Mez, Claudia L. Satizabal, Michael L. Alosco, Wei Qiao Qiu, Kathryn L. Lunetta, Joanne M. Murabito

**Affiliations:** 1 Boston University School of Public Health, Department of Biostatistics, Boston, MA 02118, USA; 2 University of Vermont, Larner College of Medicine, Department of Pathology and Laboratory Medicine, Burlington, VT 05405, USA; 3 Boston University Chobanian and Avedisian School of Medicine, Boston University Alzheimer’s Disease Research Center and CTE Center, Boston, MA 02118, USA; 4 Boston University Chobanian and Avedisian School of Medicine, Department of Neurology, Boston, MA 02118, USA; 5 Framingham Heart Study, National Heart, Lung, and Blood Institute and Boston University Chobanian and Avedisian School of Medicine, Framingham, MA 01702, USA; 6 University of Texas Health Science Center at San Antonio, Glenn Biggs Institute for Alzheimer’s and Neurodegenerative Diseases, San Antonio, TX 78229, USA; 7 Boston University Chobanian and Avedisian School of Medicine, Department of Psychiatry, Boston, MA 02118, USA; 8 Boston University Chobanian and Avedisian School of Medicine, Department of Pharmacology and Experimental Therapeutics, Boston, MA 02118, USA; 9 Boston University Chobanian and Avedisian School of Medicine, Department of Medicine, Boston, MA 02118, USA; 10 Boston Medical Center, Department of Adult Primary Care, Boston, MA 02119, USA

**Keywords:** immune cell, CMV, aging, T cells, smoking

## Abstract

Understanding the composition of circulating immune cells with aging and the underlying biologic mechanisms driving aging may provide molecular targets to slow the aging process and reduce age-related disease. Utilizing cryopreserved cells from 996 Framingham Heart Study (FHS) Offspring Cohort participants aged 40 and older (mean 62 years, 48% female), we report on 116 immune cell phenotypes including monocytes, T-, B-, and NK cells and their subtypes, across age groups, sex, cytomegalovirus (CMV) exposure groups, smoking and other cardiovascular risk factors. The major cellular differences with CMV exposure were higher Granzyme B+ cells, effector cells, and effector-memory re-expressing CD45RA (TEMRA) cells for both CD4+ and CD8+. Older age was associated with lower CD3+ T cells, lower naïve cells and naïve/memory ratios for CD4+ and CD8+. We identified many immune cell differences by sex, with males showing lower naïve cells and higher effector and effector memory cells. Current smokers showed lower pro-inflammatory CD8 cells, higher CD8 regulatory type cells and altered B cell subsets. No significant associations were seen with BMI and other cardiovascular risk factors. Our cross-sectional observations of immune cell phenotypes provide a reference to further the understanding of the complexity of immune cells in blood, an easily accessible tissue.

## INTRODUCTION

Chronic low-grade inflammation during aging (i.e., inflammaging) and immunosenescence are biologic processes associated with vulnerability to age-related diseases including neurodegenerative disorders, cardiovascular disease, diabetes, and cancer [1]. Age-related changes in the immune system may also result in the increased susceptibility to infection with pathogens such as influenza, SARS-CoV-2, and bacterial pneumonia [2, 3] and impaired vaccine response [4] observed in older adults. Chronic activation of innate immunity [5] and changes in the number and composition of circulating immune cells, including B cells, CD4+ and CD8+ T cells and their respective subsets, involved in antibody response and cell mediated immune response occur with age [6, 7]. A variety of factors influence the distribution of T cell subsets beyond age including genetics, sex [8], and infection with cytomegalovirus (CMV) [9], a herpesvirus that commonly infects older adults and remains latent or persistent for years [10]. CMV seropositivity associates with greater number of CD4 and CD8 T effector cells [11] and expansion of memory T cells including CD4 effector memory and CD4 and CD8 effector memory T cells that re-express naive cell marker (TEMRA cells) [6, 12]. This clonal expansion of CMV-specific CD4 and CD8 effector memory cells greatly diminishes the T cell repertoire to other challenges. Circulating immune cell phenotypes have been associated with cardiovascular disease traits, including subclinical atherosclerosis [13] and diabetes [14]. A better understanding of the role of age, sex, and exposure to CMV infection on immune cell phenotypes may lead to a better understanding of the contribution of these cells to cardiovascular disease and other age-related diseases.

The Health and Retirement Study (HRS), a nationally representative sample of older adults from the United States, found that the numbers of naïve CD4+ and CD8+ T cells were strongly and inversely related to age in both CMV positive and CMV negative adults and were lower in men [11]. In contrast, age-related associations with CD4+ and CD8+ effector cell subsets were dependent upon CMV serostatus [11]. The CD4+/CD8+ ratio, a widely used measure of immunosenescence, was positively correlated to age in CMV seropositive individuals [11] and associated with mortality in older adults [9, 15, 16]. Recently, composite age-related immune phenotype (ARIP) measures based on age-related changes of naïve, memory, and effector T cell subsets for CD4+ and CD8+ T cells have been proposed [17]. The CD4+ ARIP measure was found to have stronger associations with biological age, age-related morbidity, and mortality compared to other ARIP measures while the CD8+ ARIP measure was associated with specific health conditions [17]. Whether composite ARIP measures enhance the prediction of age-related disease risk beyond specific immune cell types requires further investigation.

Understanding the differences in circulating immune cells with age and the underlying biologic mechanisms driving immune cell aging may provide molecular targets to slow the aging process [18] and in turn reduce age-related disease. However, those changes in the composition of circulating immune cells particularly at the level of cell subsets within T cells, B cells, and natural killer (NK) cells is not fully documented [7]. We performed immune-profiling of a broad panel of innate and adaptive immune cell phenotypes in cryopreserved cells from dementia-free Framingham Heart Study (FHS) Offspring Cohort participants aged 40 and older. We report on 116 circulating immune cell phenotypes including T cells, B cells, NK cells, and monocytes extending the breadth of immune cell profiling in prior reports. We hypothesize that we will identify immune cell phenotype and ARIP measure associations with CMV serostatus, age, and sex, as well as associations with cardiovascular risk factors.

## RESULTS

### Summary of immune cell phenotypes profiled

**Figure 1:**
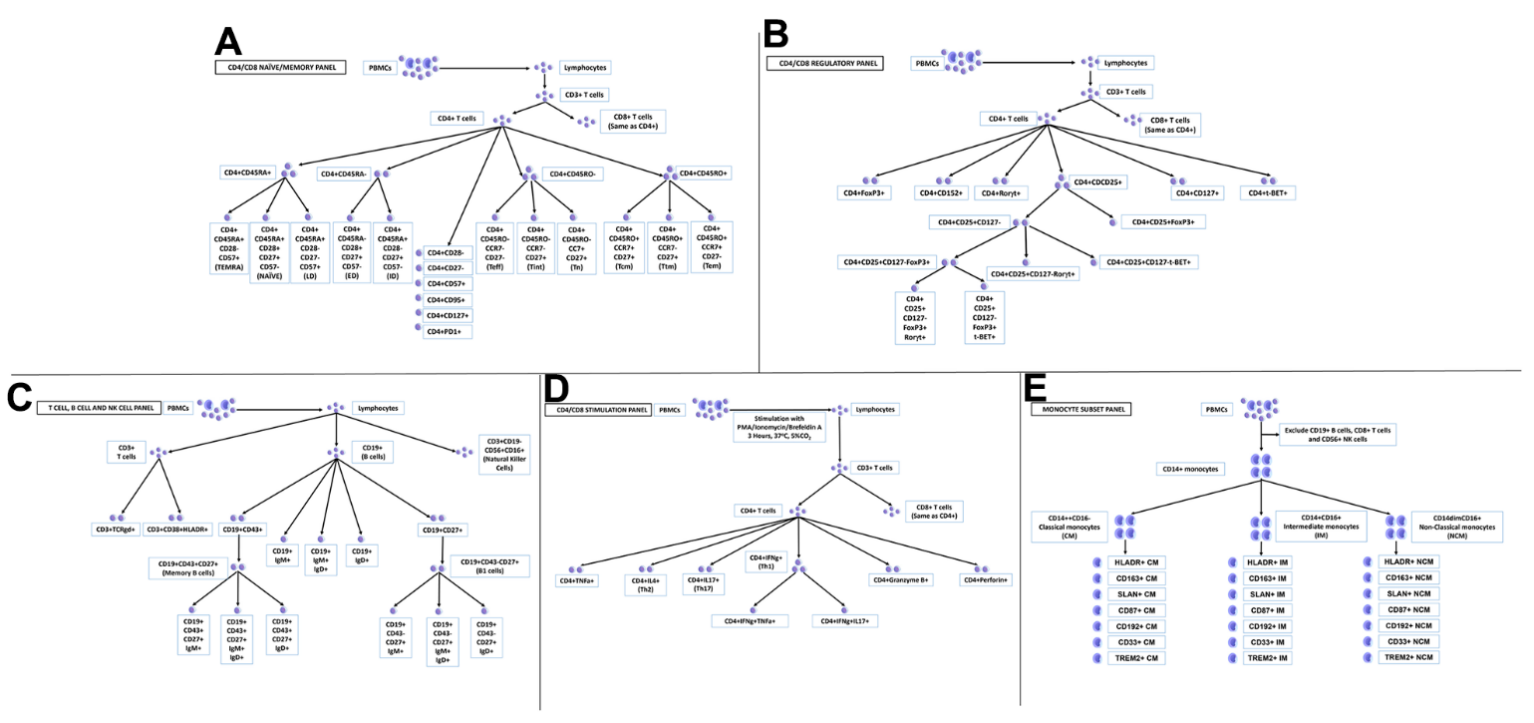
**Peripheral blood mononuclear cells (PBMCs) profiled in this study.** (**A**) CD4/CD8 naïve/memory panel; (**B**) CD4/CD8 regulatory panel; (**C**) T cell, B cell and NK cell panel; (**D**) CD4/CD8 stimulation panel; (**E**) monocyte subset panel.

Immune cell phenotypes profiled from peripheral blood mononuclear cells (PBMCs) samples are shown in Figure 1 and listed in Supplementary Table 1. We selected 24 immune cell phenotypes including the CD4+ and CD8+ naïve cells, effector cells, memory cells, senescent cells, and Granzyme B producing CD4 and CD8 cells, B cells, NK cells, classical monocytes, and the 3 ARIP measures to illustrate our main findings, with complete results for all 116 immunophenotypes for all analyses found in the online supplement. Cellular markers used to identify the 24 circulating immune cell phenotypes of primary focus are listed in Table 1. The number of participants with data and summary statistics (mean, standard deviation, min, max, and coefficients of variation (CV)) for 116 immune cell phenotypes and 3 ARIP measures are presented in Supplementary Table 2. Pairwise correlation plots are presented in Supplementary Figure 1. We note that we use “CD4” to indicate CD4+, and “CD8” to indicate CD8+ throughout.

**Table 1:** Characterization of primary immune cell phenotypes.

**Cell type**	**Marker used**	**% of**
**T cells**	CD3+	Live Lymphocytes
**CD4 T helper cells**	CD4+	CD3 T cells
**CD4 T Naïve cells (Tn)**	CD4+CD45RO-CCR7+CD27+	CD4 T helper cells
**CD4 T effector cells (Teff)**	CD4+CD45RO-CCR7-CD27-	CD4 T helper cells
**CD4 T effector-memory cells (Tem)**	CD4+CD45RO+CCR7-CD27-	CD4 T helper cells
**CD4 T central memory cells (Tcm)**	CD4+CD45RO+CCR7+CD27+	CD4 T helper cells
**CD4 TEMRA cells**	CD4+CD45RA+CD28-CD57+	CD4 T helper cells
**CD4+CD25+ cells**	CD4+CD25+	CD4 T helper cells
**CD4 FoxP3 T reg cells**	CD4+CD25+FoxP3+	CD4 T helper cells
**CD4 Th 1 cells**	CD4+IFNg+	CD4 T helper cells
**Granzyme B producing CD4+ cells**	GRANZYME B + CD4+	CD4 T helper cells
**CD8 Cytotoxic T cells**	CD8+	CD3 T cells
**CD8 T Naïve cells (Tn)**	CD8+CD45RO-CCR7+CD27+	CD8 Cytotoxic T cells
**CD8 T effector cells (Teff)**	CD8+CD45RO-CCR7-CD27-	CD8 Cytotoxic T cells
**CD8 T effector-memory cells (Tem)**	CD8+CD45RO+CCR7-CD27-	CD8 Cytotoxic T cells
**CD8 T central memory cells (Tcm)**	CD8+CD45RO+CCR7+CD27+	CD8 Cytotoxic T cells
**CD8 TEMRA cells**	CD8+CD45RA+CD28-CD57+	CD8 Cytotoxic T cells
**CD8+CD25+ cells**	CD8+CD25+	CD8 Cytotoxic T cells
**CD8 FoxP3 T reg cells**	CD8+CD25+FoxP3+	CD8 Cytotoxic T cells
**CD8 Tc 1 cells**	CD8+IFNg+	CD8 Cytotoxic T cells
**Granzyme B producing CD8+ cells**	Granzyme B+ CD8+	CD8 Cytotoxic T cells
**B cells**	CD19+	Live Lymphocytes
**NK cells**	CD3-CD56+CD16+	Live Lymphocytes
**Classical monocytes**	CD14+CD16-	CD14 monocytes
**CD4/CD8**	CD4 T helper cells / CD8 Cytotoxic T cells	--
**CD4 Tn/Tm**	CD4+ Tn / (CD4 Teff + CD4 Tem + CD4 Tcm)	--
**CD8 Tn/Tm**	CD8+ Tn / (CD8 Teff + CD8 Tem + CD8 Tcm)	--

Note: TEMRA cells, CD45RA-re-expressing T effector memory cells; T regs cells, T regulatory cells; Th 1 cells, T helper type 1 cells; Tc 1 cells, Cytotoxic T cells type 1; NK cells, Natural killer cells; Tn, naïve T cells; Tm, memory T cells; Teff, T effector cells; Tem, T effector-memory cells; Tcm, T central memory cells.

### Participant demographics

**Figure 2:**
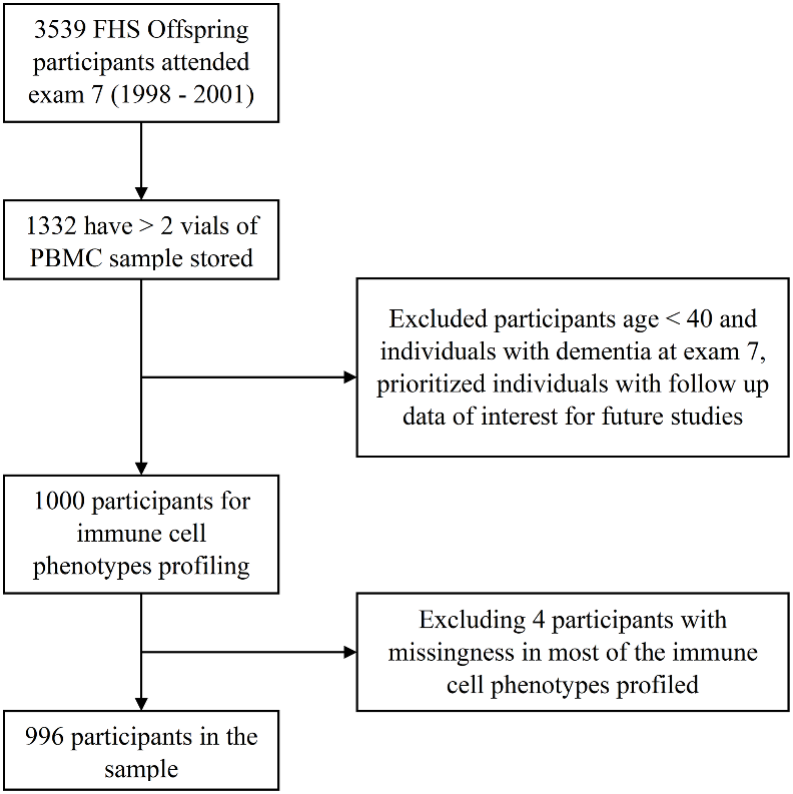
Study sample of the immune cell phenotypes in the FHS.

Sample inclusion and exclusion criteria are shown in Figure 2. Participant characteristics are summarized in Table 2. Among the 996 participants included in the study, 48% were female. The average age of the participants was 62 years with a range from 40 to 88 years. CMV values were categorized into four groups by level of CMV IgG measured in U/ml. A total of 465 participants (46.7%) were CMV seropositive, 101 of whom had values above the limit of detection (CMV > 800 U/ml). More participants had CMV levels in the equivocal range (11< CMV ≤ 15 U/ml, n=399) than in the negative range (CM ≤ 11 U/ml, n=75). CMV positive (15 < CMV ≤ 800 U/ml) participants were older on average than participants who were CMV negative or equivocal (Table 3).

**Table 2:** Participant characteristics.

**Demographic**	**All**	**Male**	**Female**
**Sample size, n (%)**	996 (100%)	516 (52%)	480 (48%)
**Age, mean (range)**	62 (40, 88)	62 (41, 85)	62 (40, 88)
**CMV positive, n (%)**	465 (46.7%)	250 (48%)	215 (45%)
***APOE-ϵ4* carriers, n (%)**	222 (22.3%)	114 (22.1%)	108 (22.5%)
***APOE-ϵ2* carriers, n (%)**	140 (14.1%)	62 (12.0%)	78 (16.3%)
**Attended college, n (%)**	733 (73.6%)	363 (70.3%)	370 (77.1%)
**Current smoker, n (%)**	139 (14%)	89 (17%)	50 (10%)
**BMI kg/m2, mean (sd)**	28 (5)	28 (6)	28 (4)
**SBP mmHg, mean (sd)**	127 (18)	126 (20)	128 (17)
**DBP mmHg, mean (sd**	74 (10)	72 (10)	76 (10)
**Hypertension Rx, n (%)**	343 (34.4%)	156 (30.2%)	187 (39.0%)
**Total cholesterol mg/dL, mean (sd)**	199 (37)	206 (37)	192 (36)
**HDL mg/dL, mean (sd)**	53 (17)	60 (16)	46 (13)
**Lipid Rx, n (%)**	225 (22.6%)	96 (18.6%)	129 (26.9%)
**Fasting blood glucose mg/dL, mean (sd)**	105 (27)	101 (26)	108 (28)
**T2DM Rx, n (%)**	61 (6.1%)	28 (5.4%)	33 (6.9%)
**Prevalent CVD, n (%)**	142 (14.3%)	46 (8.9%)	96 (20.0%)
**Prevalent AF, n (%)**	42 (4.2%)	16 (3.1%)	26 (5.4%)
**Blood cancer prevalent exam 7, n (%)**	6 (1%)	5 (1%)	1 (0%)

Notes: CMV, cytomegalovirus; *APOE*, apolipoprotein E; BMI, body mass index; sd, standard deviation; SBP, systolic blood pressure; DBP, diastolic blood pressure; Rx, medication treatment; HDL, High-density lipoprotein cholesterol; CVD, cardiovascular disease; AF, atrial fibrillation; T2DM, type II diabetes.

**Table 3:** Participants age and sex by CMV status.

	**Negative (0,11]**	**Equivocal (11,15]**	**Positive (15,800]**	**Positive, above LOD (>800)**	**Missing**
**Sample size**	75	399	364	101	57
**Female, n (%)**	36 (48%)	194 (48%)	181 (49%)	69 (68%)	36 (63%)
**Age, mean (sd)**	59 (9)	58 (7)	63 (9)	64 (8)	66 (9)
**CMV level, U/ml, median (IQR)**	10.76 (0.26)	12.18 (1.59)	208.38 (310.06)	--	--

Note: CMV, cytomegalovirus; LOD, limit of detection; IQR, inter-quartile range.

### Immune cell phenotypes across age groups by sex and CMV status

**Figure 3:**
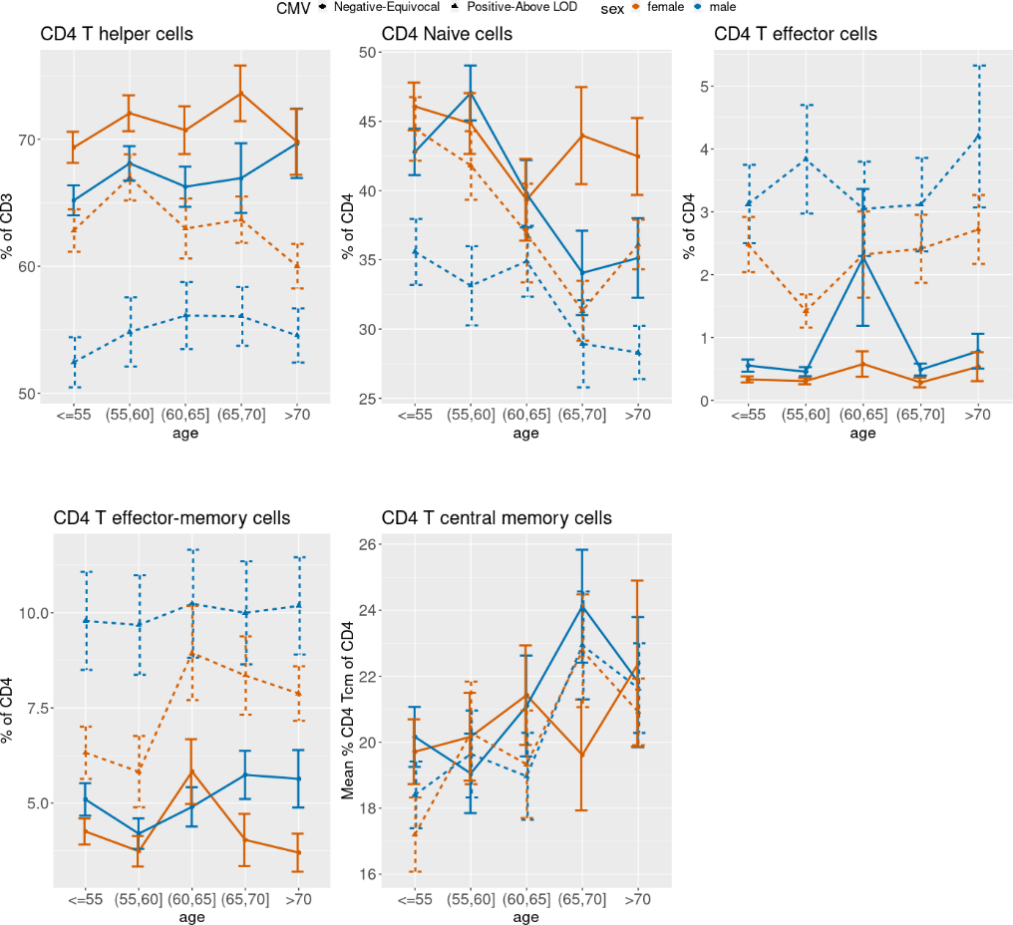
**Unadjusted mean percentages of five CD4+ T cell types by age, sex, and two-category CMV status.** Note: Teff = T effector cells; Tem = T effector-memory cells; Tcm = T central memory cells.

**Figure 4:**
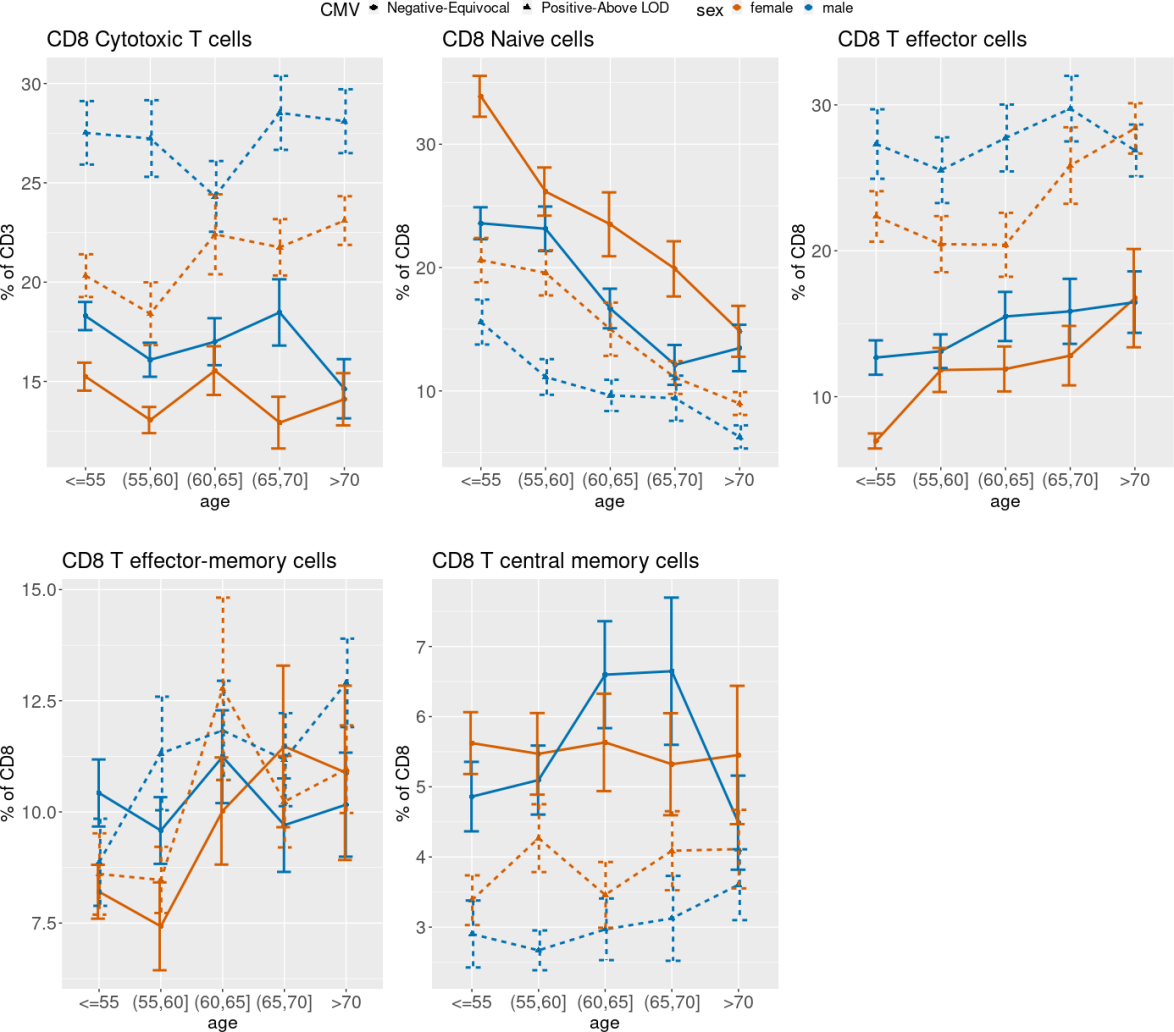
**Unadjusted mean percentages of five CD8+ T cell types by age, sex and two-category CMV status.** Note: Teff = T effector cells; Tem = T effector-memory cells; Tcm = T central memory cells.

Figure 3 (CD4), 4 (CD8) and 5 (ARIP) show the unadjusted percentages of immune cell types CD4 and CD8 and subtypes naïve (Tn), effector (Teff), effector memory (Tem) and central memory (Tcm) across age groups, stratified by sex and CMV status (as two categories by grouping the negative and equivocal, and the positive and above LOD). The percentage of CD4 T helper cells was higher in CMV negative and equivocal participants compared to CMV positive participants across all age groups and for both males and females (Figure 3), while the percentage of CD8 cytotoxic T cells in participants in the negative and equivocal CMV group was lower than in CMV positive group (Figure 4). Females had higher percentages of CD4 T helper cells and lower percentages of CD8 cytotoxic cells than males for most age and CMV serostatus groups.

Figure 3 shows that CD4 Tn cell percentages were lower in older age groups compared to younger age groups, while CD4 Tcm percentages were higher in older age groups compared to younger age groups. Both age trends were similar for CMV positive and negative male and female subgroups. Female participants in the CMV positive groups tended to have higher CD4 Tn percentages than CMV positive male participants in all age groups. Percentages of CD4 Teff cells and CD4 Tem cells were higher in CMV positive participants than negative/equivocal participants across all age groups, and males had higher proportions of these cells than females within CMV category for most age groups.

CD8 Tn cell percentages were lower in older age groups compared to younger, while CD8 Teff and Tem cell percentages were higher in older age groups compared to younger, and CD8 Tcm cells did not show a consistent age trend within sex and CMV strata (Figure 4). Percentages of CD8 Teff were higher in CMV positive participants compared to CMV negative/equivocal and were higher in males than females within each CMV group across all but the oldest age group. CD8 Tcm percentages were lower in CMV positive compared to negative/equivocal; males in the CMV positive group had higher percentages than females in all age groups.

**Figure 5:**
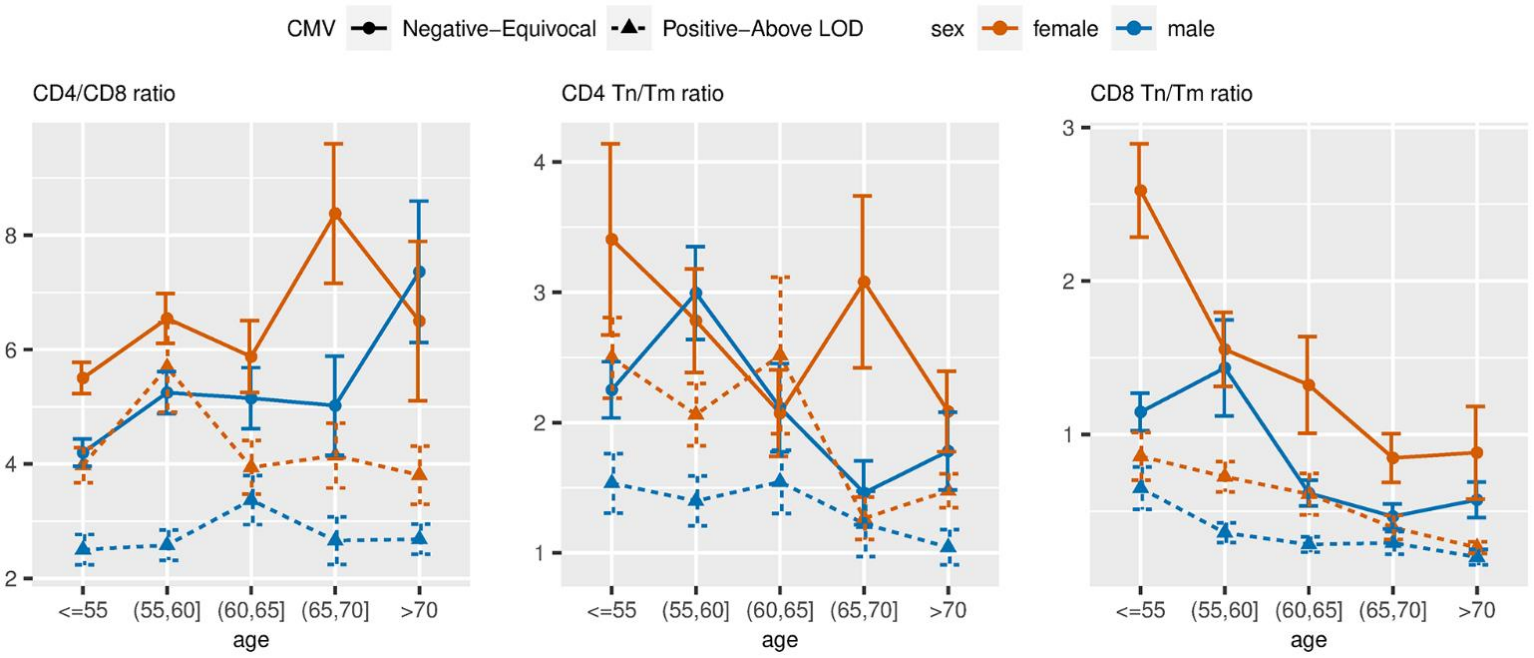
**Unadjusted mean values of ARIP measures stratified by age, sex and two-category CMV status.** Note: Tn = naïve T cells; Tm = memory T cells.

On average, we observed a higher CD4/CD8 ratio and higher CD8 Tn/Tm ratio in the CMV negative/equivocal group compared to the CMV positive groups within each sex stratum, and higher levels in females compared to males within each CMV group. The CD4/CD8 ratio was higher in the older age groups for the CMV negative/equivocal participant group but was similar across age groups for the CMV positive groups (Figure 5). Both CD4 and CD8 Tn/Tm ratios were lower in older age groups compared to younger age groups regardless of sex or CMV serostatus, but the CD4 Tn/Tm ratio fluctuated more by age group than the CD8 Tn/Tm ratio. The unadjusted percentages of other immune cell types listed in Table 1 across age group, sex, and CMV exposure groups are illustrated in Supplementary Figure 2.

### Association of immune cell phenotypes with CMV serostatus groups adjusting for age and sex

Table 4 shows that among the 27 immune cell phenotypes listed in Table 1, 20 had significantly different means across the four CMV serostatus groups after adjusting for age and sex at false discovery rate (FDR)<0.05. For most cell types, effects were not linear with increasing CMV value group, but the non-linear patterns were not the same for all cell types. For most of the significant cell types, the Equivocal CMV and Negative CMV group adjusted means were more like each other than the Above LOD group and the Positive CMV group adjusted means. We observed that CD3+ T cell levels (“T cells” in Table 4) were similar for CMV negative and equivocal participant groups but were higher in CMV positive and higher yet in the above LOD CMV positive participant groups. CD4 T helper cell levels were slightly higher for participants in the CMV equivocal group compared to those participants who were CMV negative, while the CMV positive and above LOD groups had substantially lower levels than the negative group. CD8 cytotoxic T cell levels were lower in CMV equivocal compared to CMV negative groups, and higher in the CMV positive and LOD groups compared to the negative group.

**Table 4:** Association of immune cell phenotypes with CMV status adjusting for age and sex.^a^

	**Negative (0,11] U/ml**	**Equivocal (11,15] U/ml**	**Positive (15,800]**	**Above LOD (>800)**	**FDR**
	**n=75**	**n=399**	**n=364**	**n=101**	
**T cells**	ref	-0.01 (0.12)	0.25 (0.12)	0.50 (0.15)	**1.46e-05**
**CD4 T helper cells**	ref	0.17 (0.12)	-0.52 (0.12)	-0.72 (0.14)	**8.71e-26**
**CD4 T Naïve cells (Tn)**	ref	0.19 (0.12)	-0.18 (0.12)	-0.22 (0.15)	**2.20e-06**
**CD4 T effector cells (Teff)**	ref	0.05 (0.11)	0.86 (0.11)	1.39 (0.13)	**7.79e-52**
**CD4 T effector-memory cells (Tem)**	ref	-0.11 (0.12)	0.48 (0.12)	0.78 (0.14)	**1.74e-21**
CD4 T central memory cells (Tcm)	ref	-0.21 (0.13)	-0.22 (0.13)	-0.45 (0.15)	5.53e-02
**CD4 TEMRA cells**	ref	0.07 (0.10)	0.94 (0.11)	1.42 (0.13)	**2.59e-60**
CD4 +CD25+ cells	ref	-0.08 (0.13)	-0.16 (0.13)	-0.06 (0.16)	6.25e-01
CD4 FoxP3 T reg cells	ref	0.01 (0.12)	0.10 (0.12)	0.12 (0.15)	6.64e-01
**CD4 Th 1 cells**	ref	-0.05 (0.12)	0.59 (0.12)	0.95 (0.14)	**7.79e-27**
**Granzyme B producing CD4+ cells**	ref	-0.11 (0.10)	0.98 (0.10)	1.58 (0.12)	**1.65e-93**
**CD8 Cytotoxic T cells**	ref	-0.21 (0.11)	0.60 (0.12)	0.79 (0.14)	**1.77e-35**
**CD8 T Naïve cells (Tn)**	ref	0.10 (0.10)	-0.52 (0.10)	-0.72 (0.12)	**5.96e-29**
**CD8 T effector cells (Teff)**	ref	-0.02 (0.11)	0.81 (0.11)	1.14 (0.13)	**1.21e-47**
**CD8 T effector-memory cells (Tem)**	ref	-0.30 (0.12)	-0.11 (0.13)	-0.31 (0.15)	**2.34e-02**
**CD8 T central memory cells (Tcm)**	ref	0.05 (0.12)	-0.52 (0.12)	-0.68 (0.15)	**6.86e-17**
**CD8 TEMRA cells**	ref	0.04 (0.11)	0.86 (0.11)	1.06 (0.13)	**4.57e-42**
CD8+CD25+ cells	ref	-0.08 (0.13)	-0.10 (0.13)	-0.00 (0.15)	8.31e-01
CD8 FoxP3 T reg cells	ref	0.09 (0.12)	0.08 (0.12)	0.08 (0.15)	9.37e-01
**CD8 Tc 1 cells**	ref	-0.06 (0.12)	0.38 (0.12)	0.56 (0.15)	**6.73e-11**
**Granzyme B producing CD8+ cells**	ref	-0.15 (0.10)	0.75 (0.10)	1.03 (0.13)	**3.27e-53**
**B cells**	ref	0.03 (0.13)	-0.20 (0.13)	-0.37 (0.15)	**1.93e-03**
NK cells	ref	0.09 (0.12)	-0.08 (0.12)	-0.10 (0.15)	1.79e-01
Classical monocytes	ref	0.04 (0.13)	0.07 (0.13)	-0.15 (0.16)	4.35e-01
**CD4/CD8**	ref	0.22 (0.11)	-0.58 (0.12)	-0.77 (0.14)	**7.86e-34**
**CD4 Tn/Tm**	ref	0.17 (0.12)	-0.24 (0.12)	-0.31 (0.15)	**3.86e-08**
**CD8 Tn/Tm**	ref	0.15 (0.10)	-0.57 (0.10)	-0.80 (0.12)	**3.58e-38**

Note: TEMRA cells, CD45RA-re-expressing T effector memory cells; T regs cells, T regulatory cells; Th 1 cells, T helper type 1 cells; Tc 1 cells, Cytotoxic T cells type 1; NK cells, Natural killer cells; Tn, naïve T cells; Tm, memory T cells.

^a^Complete case data, immune cell phenotypes were inverse normal transformed. Table values are difference in adjusted mean value (in standard deviation units) from the reference (CMV negative) group. The threshold for declaring significance was FDR < 0.05.

Among the CD4 cell subtypes, the CMV positive and above LOD groups had lower age and sex adjusted CD4 Tn levels than the CMV negative or equivocal groups, while adjusted Teff, Tem, TEMRA, T helper type 1 (Th 1) and Granzyme B producing cell subtype levels were higher in the CMV positive and above LOD groups than in the negative and equivocal groups. We observed similar patterns among the corresponding CD8 cell subtypes, the one exception being that CD8 Tcm cells also differed by CMV status.

B cell levels were similar in negative and equivocal CMV groups, and lower in the CMV positive groups. Finally, all three ARIP measures tested were significantly different across the CMV groups, with slightly higher ratios in the CMV equivocal group compared to the negative group, and lower ratios in the CMV positive and above LOD participant groups compared to the negative group.

The age and sex adjusted CMV associations for all 119 immune cell phenotypes for individuals with CMV measurement are presented in Supplementary Table 4. Among the 119 total immune cell phenotypes and ARIP measures, 63 had significantly different levels across the four CMV serostatus groups (Supplementary Table 4); results were similar when including “Missing” as an additional CMV category (Supplementary Table 5). We tested for interactions between age and CMV, and between sex and CMV, but did not observe significant interactions (data not shown).

### Association of immune cell phenotypes with age group adjusting for multiple covariates

As shown in Table 5, seven of the 27 primary immune cell phenotypes listed in Table 1 differ across the 5 age groups after adjusting for the “Model 2” covariates described in Methods at FDR<0.05. The age effect did not appear to be linear with the increasing age groups for most of the seven significant cell types.

**Table 5:** Association of immune cell phenotypes with age adjusting for Model 2 covariates.^a^

	**≤55 (n=286)**	**56-60 (n=208)**	**61-65 (n=166)**	**66-70 (n=140)**	**≥71 (n=196)**	**FDR**
**T cells**	ref	-0.01 (0.09)	-0.23 (0.10)	-0.33 (0.11)	-0.35 (0.10)	**9.34e-03**
CD4 T helper cells	ref	0.23 (0.08)	0.13 (0.09)	0.23 (0.10)	0.15 (0.10)	2.31e-01
**CD4 T Naïve cells (Tn)**	ref	0.01 (0.09)	-0.21 (0.10)	-0.41 (0.11)	-0.33 (0.10)	**2.01e-03**
CD4 T effector cells (Teff)	ref	-0.06 (0.08)	0.07 (0.08)	-0.07 (0.09)	-0.00 (0.09)	8.01e-01
CD4 T effector-memory cells (Tem)	ref	-0.13 (0.08)	0.08 (0.09)	0.10 (0.10)	0.08 (0.10)	3.49e-01
CD4 T central memory cells (Tcm)	ref	0.03 (0.09)	0.11 (0.10)	0.30 (0.11)	0.22 (0.11)	2.37e-01
CD4 TEMRA cells	ref	-0.06 (0.07)	0.00 (0.08)	-0.03 (0.09)	-0.04 (0.09)	9.50e-01
CD4+CD25+ cells	ref	0.07 (0.09)	0.00 (0.10)	-0.07 (0.11)	0.04 (0.11)	8.92e-01
CD4 FoxP3 T reg cells	ref	0.00 (0.09)	0.06 (0.10)	0.03 (0.11)	0.02 (0.11)	9.79e-01
CD4 Th 1 cells	ref	-0.00 (0.08)	0.06 (0.09)	0.08 (0.10)	0.00 (0.10)	9.50e-01
Granzyme B producing CD4+ cells	ref	0.00 (0.07)	0.08 (0.07)	0.07 (0.08)	0.08 (0.08)	8.49e-01
CD8 Cytotoxic T cells	ref	-0.23 (0.08)	-0.15 (0.09)	-0.12 (0.10)	-0.17 (0.09)	2.53e-01
**CD8 T Naïve cells (Tn)**	ref	-0.17 (0.07)	-0.45 (0.08)	-0.63 (0.09)	-0.84 (0.09)	**1.89e-19**
**CD8 T effector cells (Teff)**	ref	0.14 (0.07)	0.18 (0.08)	0.34 (0.09)	0.33 (0.09)	**1.02e-02**
CD8 T effector-memory cells (Tem)	ref	0.02 (0.09)	0.29 (0.10)	0.19 (0.11)	0.26 (0.11)	9.30e-02
CD8 T central memory cells (Tcm)	ref	0.10 (0.09)	0.15 (0.09)	0.09 (0.10)	0.07 (0.10)	8.04e-01
CD8 TEMRA cells	ref	0.08 (0.08)	0.07 (0.08)	0.23 (0.09)	0.26 (0.09)	1.68e-01
CD8+CD25+ cells	ref	-0.05 (0.09)	-0.01 (0.10)	-0.13 (0.11)	0.08 (0.11)	7.11e-01
CD8 FoxP3 T reg cells	ref	-0.01 (0.09)	-0.14 (0.10)	-0.06 (0.10)	-0.11 (0.10)	8.04e-01
CD8 Tc 1 cells	ref	0.13 (0.09)	0.21 (0.10)	0.26 (0.10)	0.24 (0.10)	2.64e-01
**Granzyme B producing CD8+ cells**	ref	0.12 (0.07)	0.22 (0.08)	0.26 (0.09)	0.34 (0.09)	**1.35e-02**
B cells	ref	0.02 (0.09)	-0.10 (0.10)	0.15 (0.11)	0.12 (0.11)	5.33e-01
NK cells	ref	-0.01 (0.09)	0.19 (0.10)	0.21 (0.11)	0.25 (0.11)	1.98e-01
Classical monocytes	ref	-0.12 (0.09)	0.03 (0.10)	-0.04 (0.11)	-0.14 (0.11)	7.07e-01
CD4/CD8	ref	0.25 (0.08)	0.16 (0.09)	0.17 (0.10)	0.17 (0.10)	1.86e-01
**CD4 Tn/Tm**	ref	0.03 (0.09)	-0.18 (0.10)	-0.36 (0.11)	-0.29 (0.10)	**9.34e-03**
**CD8 Tn/Tm**	ref	-0.14 (0.07)	-0.43 (0.08)	-0.58 (0.09)	-0.75 (0.09)	**1.78e-16**

Note: TEMRA cells, CD45RA-re-expressing T effector memory cells; T regs cells, T regulatory cells; Th 1 cells, T helper type 1 cells; Tc 1 cells, Cytotoxic T cells type 1; NK cells, Natural killer cells; Tn, naïve T cells; Tm, memory T cells.

^a^Immune cell phenotypes were inverse normal transformed. Table values are difference in adjusted mean value (in standard deviation units) from the reference (age ≤ 55 group. Model 2 covariates include sex, logCMV, education level, APOE genotypes, BMI groups, and indicators of current smoking, presence of diabetes, prevalent CVD, prevalent AF, SBP, on hypertension treatment at exam 7, TC, HDL, on lipid treatment at exam 7, and cancer prior to exam 7. The threshold for declaring significance was FDR < 0.05.

CD3+ T cell levels were lower for each increasing age group. CD4 Tn cell levels were the only CD4 cell subtype with significant differences by age: levels were lower in the older groups compared to the younger. The two youngest age groups differed by only 0.01 SD unit, and the two oldest age group means differed by only 0.02 SD units. CD8 Tn, Teff, and Granyzme B producing CD8 cells all differed by age group at FDR<0.05. CD8 Tn cell levels were lower in each increasing age group, while the other two cell types had higher levels in older age groups.

The adjusted mean CD4 Tn/Tm ratio was similar in the two younger age groups, and lower in the three older age groups. The CD8 Tn/Tm ratio had larger differences across age groups than the CD4 Tn/Tm ratio, and the ratio was lower in each subsequent increasing age group. Together, these data are consistent with an age-related shift from naïve cells to a more senescent/exhausted phenotype, with the shift occurring in CD8 cells more evident than CD4 cells.

The multivariable adjusted associations with age groups for all 119 immune cell phenotypes are presented in Supplementary Table 6. Significant associations were seen, particularly in the CD8 compartment that are consistent with immune exhaustion from repeated replication (ie loss of CD28, CD27 and increase in CD57 expression).

### Association of immune cell phenotypes with sex adjusting for multiple covariates

Table 6 shows that 18 of the 27 primary cell types differed by sex at FDR<0.05, adjusting for Model 2 covariates. Males had lower percentages of CD4 T helper cells and higher percentages of CD8 cytotoxic cells than females. Among the CD4 and CD8 subtypes, males had lower percentages than females of both CD4 and CD8 Tn cells, as well as CD8 Tcm cells. Males had higher percentages than females of both CD4 and CD8 Teff, Tem, TEMRA, and Granzyme B producing cells. Males also had a significantly higher percentage of NK cells and significantly lower percentages of B cells compared to females. The CD4/CD8 ratio and both the CD4 and CD8 Tn/Tm ratios were lower in males compared to females.

**Table 6:** Association of immune cell phenotypes with sex adjusting for Model 2 covariates.^a^

	**Female (n=516)**	**Male (n=480)**	**FDR**
T cells	ref	-0.16 (0.07)	6.89e-02
**CD4 T helper cells**	ref	-0.40 (0.07)	**5.55e-08**
**CD4 T Naïve cells (Tn)**	ref	-0.20 (0.07)	**1.65e-02**
**CD4 T effector cells (Teff)**	ref	0.24 (0.06)	**6.58e-04**
**CD4 T effector-memory cells (Tem)**	ref	0.24 (0.07)	**2.02e-03**
CD4 T central memory cells (Tcm)	ref	0.01 (0.07)	9.17e-01
**CD4 TEMRA cells**	ref	0.17 (0.06)	**1.94e-02**
CD4+CD25+ cells	ref	-0.00 (0.07)	9.24e-01
CD4 FoxP3 T reg cells	ref	-0.08 (0.07)	4.86e-01
CD4 Th 1 cells	ref	-0.10 (0.07)	3.40e-01
**Granzyme B producing CD4+ cells**	ref	0.25 (0.05)	**1.92e-05**
**CD8 Cytotoxic T cells**	ref	0.39 (0.06)	**2.41e-08**
**CD8 T Naïve cells (Tn)**	ref	-0.41 (0.06)	**2.31e-10**
**CD8 T effector cells (Teff)**	ref	0.27 (0.06)	**5.48e-05**
**CD8 T effector-memory cells (Tem)**	ref	0.25 (0.07)	**3.44e-03**
**CD8 T central memory cells (Tcm)**	ref	-0.18 (0.07)	**3.29e-02**
**CD8 TEMRA cells**	ref	0.36 (0.06)	**7.76e-08**
CD8+CD25+ cells	ref	-0.06 (0.07)	5.87e-01
CD8 FoxP3 T reg cells	ref	0.08 (0.07)	4.36e-01
CD8 Tc 1 cells	ref	0.12 (0.07)	2.14e-01
**Granzyme B producing CD8+ cells**	ref	0.38 (0.06)	**2.53e-09**
**B cells**	ref	-0.24 (0.07)	**3.44e-03**
**NK cells**	ref	0.25 (0.07)	**2.06e-03**
Classical monocytes	ref	0.02 (0.07)	8.67e-01
**CD4/CD8**	ref	-0.41 (0.06)	**1.32e-08**
**CD4 Tn/Tm**	ref	-0.19 (0.07)	**2.68e-02**
**CD8 Tn/Tm**	ref	-0.41 (0.06)	**1.91e-10**

Note: TEMRA cells, CD45RA-re-expressing T effector memory cells; T regs cells, T regulatory cells; Th 1 cells, T helper type 1 cells; Tc 1 cells, Cytotoxic T cells type 1; NK cells, Natural killer cells; Tn, naïve T cells; Tm, memory T cells.

^a^Immune cell phenotypes were inverse normal transformed. Table values are difference in adjusted mean value (in standard deviation units) for males compared to the reference (female) group. Model 2 covariates include age, logCMV, education level, APOE genotypes, BMI groups, and indicators of current smoking, presence of diabetes, prevalent CVD, prevalent AF, SBP, on hypertension treatment at exam 7, TC, HDL, on lipid treatment at exam 7, and cancer prior to exam 7. The threshold for declaring significance was FDR < 0.05.

Sex associations for all 119 total immune cell phenotypes and ARIP measures are presented in Supplementary Table 7. Significant associations were observed that are consistent with male participants having higher levels of immune activation/exhaustion than female participants.

### Association of immune cell phenotypes with smoking status adjusting for multiple covariates

Table 7 shows that four of the 27 primary immune cell phenotypes and ARIP measures differed significantly (FDR<0.05) between current smokers and non- smokers (former or never smokers) after adjusting for Model 2 covariates. The percentages of CD8 TEMRA, Tc1, and Granzyme B producing CD8 cells were significantly lower in smokers compared to non-smokers, while CD8 FoxP3 T regulatory cell percentages were higher in smokers compared to non-smokers. It is worth noting that, while the FoxP3 transcription factor in CD25+ cells is well-known to be a regulatory cell for CD4+ cells, less is known about the true function of CD25+FoxP3+ CD8 cells. Smoking status was not significantly associated with the three ARIP measures.

**Table 7:** Association of immune cell phenotypes with smoking status adjusting for Model 2 covariates.^a^

	**Current non-smoker (n=857)**	**Current smoker (n=139)**	**FDR**
T cells	ref	0.10 (0.09)	5.34e-01
CD4 T helper cells	ref	-0.04 (0.09)	8.24e-01
CD4 T Naïve cells (Tn)	ref	-0.07 (0.09)	6.70e-01
CD4 T effector cells (Teff)	ref	0.02 (0.08)	8.66e-01
CD4 T effector-memory cells (Tem)	ref	-0.10 (0.09)	5.00e-01
CD4 T central memory cells (Tcm)	ref	0.14 (0.09)	3.28e-01
CD4 TEMRA cells	ref	0.07 (0.07)	5.61e-01
CD4+CD25+ cells	ref	-0.09 (0.09)	5.61e-01
CD4 FoxP3 T reg cells	ref	0.09 (0.09)	5.61e-01
CD4 Th 1 cells	ref	-0.12 (0.09)	3.74e-01
Granzyme B producing CD4+ cells	ref	-0.03 (0.07)	8.32e-01
CD8 Cytotoxic T cells	ref	0.12 (0.08)	3.38e-01
CD8 T Naïve cells (Tn)	ref	-0.04 (0.08)	7.63e-01
CD8 T effector cells (Teff)	ref	-0.18 (0.08)	8.20e-02
CD8 T effector-memory cells (Tem)	ref	0.05 (0.09)	7.77e-01
CD8 T central memory cells (Tcm)	ref	0.18 (0.09)	1.38e-01
**CD8 TEMRA cells**	ref	-0.24 (0.08)	**2.33e-02**
CD8+CD25+ cells	ref	-0.09 (0.09)	5.76e-01
**CD8 FoxP3 T reg cells**	ref	0.28 (0.09)	**1.65e-02**
**CD8 Tc 1 cells**	ref	-0.35 (0.09)	**2.30e-03**
**Granzyme B producing CD8+ cells**	ref	-0.21 (0.07)	**3.37e-02**
B cells	ref	0.02 (0.09)	8.84e-01
NK cells	ref	-0.24 (0.09)	5.05e-02
Classical monocytes	ref	-0.14 (0.09)	3.38e-01
CD4/CD8	ref	-0.08 (0.08)	5.47e-01
CD4 Tn/Tm	ref	-0.02 (0.09)	8.66e-01
CD8 Tn/Tm	ref	0.01 (0.07)	8.87e-01

Note: TEMRA cells, CD45RA-re-expressing T effector memory cells; T regs cells, T regulatory cells; Th 1 cells, T helper type 1 cells; Tc 1 cells, Cytotoxic T cells type 1; NK cells, Natural killer cells; Tn, naïve T cells; Tm, memory T cells.

^a^Immune cell phenotypes were inverse normal transformed. Table values are difference in adjusted mean value (in standard deviation units) from the reference (current non-smoker) group. Model 2 covariates include age, sex, logCMV, education level, APOE genotypes, BMI groups, and indicators presence of diabetes, prevalent CVD, prevalent AF, SBP, on hypertension treatment at exam 7, TC, HDL, on lipid treatment at exam 7, and cancer prior to exam 7.

Supplementary Table 8 summarizes the effect of smoking status for all 119 immune cell phenotypes; 20 of the 119 phenotypes were significantly different (FDR<0.05). Notably, although smoking status was not significantly associated with B cell levels, 8 of the B cell subtypes were significantly different in smokers compared to non-smokers. While the memory B cells were higher with smoker, the IgM+ and IgD+IgM+ memory cells were lower with smoking. (Supplementary Table 8).

### Associations of immune cell phenotypes with other variables

Adjusting for Model 2 covariates, we did not observe significant associations of the 116 immune cell phenotypes and the 3 ARIP measures with education, *APOE* genotype, obesity status, type 2 diabetes, prevalent CVD or AF at exam 7, hypertension treatment, lipid treatment, SBP, TC, and HDL levels, or diagnosis of chronic blood cancer prior to exam 7 (all FDR>0.05). Estimated effect sizes for each group, *p*–values, and FDRs are reported in Supplementary Tables 9–18.

## DISCUSSION

Among nearly 1000 Framingham Offspring cohort middle-aged and older adult men and women we characterized the circulating innate and adaptive immune system by profiling 116 immune cell phenotypes including subtypes of CD4 and CD8 T cells, B cells, NK cells, and monocytes, and calculated three ARIP measures based on T cell subtypes. We confirmed and extended known associations with CMV, age, and sex in models adjusting for important covariates. Finally, we identified associations of specific immune cell phenotypes with smoking status but not with other cardiovascular disease risk factors and cardiovascular disease. Thus, these factors, including hypertension, cardiovascular disease, atrial fibrillation, and diabetes, as well as body mass index, lipid treatment, systolic blood pressure, total cholesterol, and high-density cholesterol levels, are unlikely to confound the associations between immune cell levels and age, sex, CMV status, and smoking status that we observed in this study, or associations with outcomes of interest that will be explored in future studies. Our cross-sectional observations of immune cell phenotypes in a large community sample provide a reference for the scientific community to further our understanding of the complexity of the immune cell subsets and diversity of the immune system in the blood, a tissue that is easily accessible in human population studies.

As expected nearly half the participants in our study sample were CMV positive with women making up about two-thirds of the CMV positive group above LOD. Viral infection, particularly from CMV, is known to associate with alterations to the immune system [10, 12, 19], making it critical to investigate the influence of CMV exposure on specific immune cell phenotypes when examining immune cells in population studies. Overall, we observed 63 immune cell phenotypes associated with CMV serostatus including differences in CD3+ T cells and T cell subsets, with similar findings in men and women and some findings independent of age-related differences. CD3+ T cell levels were higher in CMV positive groups compared to CMV negative/equivocal groups. CD4 and CD8 Tn cells were lower in the CMV positive group and had similar associations with age and sex but were lower in men. The CD4 and CD8 TEMRA and granzyme B producing cells were higher in the CMV positive group in both men and women, with higher levels in men. Only the CD8 granzyme B producing cells were age-related. CD4 Teff, CD4 Tem, and CD8 Teff were higher in CMV positive individuals while CD8 Tem, and CD8 Tcm were lower. Consistent with a previous report [20], Th1 cells were higher with CMV positivity. Additionally, T-bet+ CD4+ cells, which regulate Th1 differentiation, were also higher with CMV positivity. All ARIP measures were lower for the CMV positive groups compared to negative and equivocal groups consistent with known expansion of effector and memory T cell subsets in the presence of CMV. Future investigations of the association of ARIP indicators with age-related outcomes will require adjustment for CMV status, depending on outcome of interest.

Our findings largely confirm other studies that identified increases in memory and TEMRA T cell subsets with CMV positive status [12, 21, 22]. However, we observed lower levels of several memory subsets (CD8 Tcm, and CD8 Tem) in CMV positive individuals in contrast to another study that observed higher levels of these T cell subsets in CMV positive individuals or no difference by CMV serostatus [11]. These differences are likely due to different cell surface markers used to identify these subsets (most notably CD27 versus CD28) or the method of quantification (percentages vs cell counts). All ARIP measures were lower for CMV positive individuals compared to negative and equivocal groups. Our study also observed an association of CMV status with B cells. Reports in the literature have been inconsistent with one study identifying minimal affects [23] and another failing to observe an association of B cells with CMV [12]. Consistent with a prior report [24], classical monocytes did not differ by CMV group, nor did we observe associations with age group or sex. Differences in this case may also be due to markers used to identify subsets and quantification methods utilized. Our data are consistent with CMV positive individuals having a shift from an immunologically naïve phenotype towards an exhausted pro-inflammatory phenotype, characterized by markers of replicative senescence (loss of CD27, CD23, higher CD57) and higher production of Granzyme B, perforin, and interferon gamma.

We expand upon a recent report from the Health and Retirement Study of T cells, CD4 and CD8 cells and their naïve, effector, effector memory, and central memory subtypes [11] to include additional CD4 and CD8 subtypes and other immune cell phenotypes (B cells, NK cells and monocytes). Our study identified 18 immune cell phenotypes associated with age including in the naïve, effector, and memory subtypes with most results observed for CD8 T cell subtypes. Age-related associations were observed after adjustment for CMV status, sex, and other covariates. We did not observe an association between the CD4/CD8 ratio with age but did find a negative association between two ARIP phenotypes, CD4 Tn/Tm and CD8 Tn/Tm ratio with age. These ARIP phenotypes may prove important in establishing meaningful differences in chronological and biological age. Studies using absolute cell counts have demonstrated that CD4 Tn/Tm and CD4 Tn are strongly associated with clinically-defined biological age, while CD8 Tn/Tm and CD8 Tn are strongly correlated with chronological age [17]. Interestingly, it was the phenotypes that correlated with biological age, namely CD4 Tn/Tm and CD4 Tn, that were also associated with multimorbidity, cancer, diabetes and heart disease. This may be due to the method of assigning biological age that utilizes biomarkers of cardiovascular, metabolic, inflammation as well as liver, lung and kidney function. Our finding that men have lower CD4 and CD8 Tn and Tn/Tm than women may explain some of the differences observed in the healthspan and lifespan in men vs. women. We did not observe age associations for B cells, NK cells or classical monocytes in contrast to Milieu Interieur cohort [12] and a smaller study that noted age-related correlations in all of these immune cell phenotypes [25]. One potential reason for differences in associations may be due to the differences in age distribution of our study and the former study [25] that investigated young adults (ages 20-31 years) and older adults (ages 60-96 years) with both young and older ages beyond the age ranges of our study sample. Additionally, the Milieu Interieur cohort utilized cell counts or mean fluorescent intensity to quantitate cells, while the smaller study used absolute cell counts, both in contrast to our study which used the percentages of major parent (i.e. CD4 for CD4 subsets). We chose percentages as a means to visualize the composition of the major phenotypes (i.e. CD4 or CD8 cells) without the influence of overall white cell counts which can vary greatly within the population.

Consistent with previous reports [11, 16, 19], in adjusted models we observed that CD3+ T cells and CD4 and CD8 Tn cells were lower in older age-groups. Tn cells provide defense against new antigens and infections such as SARS-CoV-2. Lower T cell subsets correlated with adverse COVID-19 outcomes and may in part explain the higher susceptibility to COVID-19 with age [26–28]. CD8 effector subsets were higher with increasing age after adjusting for CMV serostatus. Effector cell subsets are known to provide protection against previously encountered infections and antigens. Our observations add to prior reports that have shown an increase in CD8 Teff cells with age [25] although results in the literature have been inconsistent with reports of associations with age only in CMV positive individuals [19] or lack of age associations [11]. In addition, we report associations between Granzyme B producing CD8 cells with increasing age. A small study (n=28) of healthy young (ages 25-29) and old (ages 62-70) non-obese men did not observe higher Granzyme B producing CD8 cells in older men but did show a trend toward higher levels of this cell population in the older participants [29]. Their data shows that the granzyme B is being produced primarily by CD57+ cells, indicating replicative senescence [30]. Additionally, Granzyme B production in both CD4 and CD8 cells is increased with CMV seropositivity which increases with age [31]. While we adjusted for this in our models, the granzyme B producing CD8 cells were still associated with age. This data may indicate an important role for Granzyme B production in increasing the cytotoxicity of CD8+ cells with age, particularly in CMV positive individuals, where decreased immune-repertoire may be replaced by highly cytotoxic Granzyme B producing cells [32].

There are recognized differences in the immune system in men and women with women at greater risk for autoimmune disease. Differences increase at older ages (after age 65 years), with men observed to have higher levels of innate activity (inflammaging) and lower adaptive immunity compared to women [33], although both men and women were observed to have these immune system changes with age. We observed adverse levels throughout the T cell repertoire after adjusting for CMV, age and other covariates in men compared with women including lower Tn, higher Tem, higher TEMRA and higher granzyme B producing T cells in both CD4 and CD8 subsets. ARIPs including the CD4/CD8 ratio were also lower in men compared with women. A smaller study of healthy Japanese adults with more limited immune cell profiling also observed more favorable age-related changes in immune parameters in women compared men [8] as did the Milieu Interieur Cohort study [12]. The Health and Retirement Study reported sex differences, with men observed to have lower CD4 and CD8 Tn cells and higher percent of male participants with low CD4/CD8 ratio [11], similar to our findings.

Finally, we also observed lower levels of B cells in men compared to women consistent with other reports [8, 33] and higher levels of NK cells, as previously reported [34]. B cells produce antibodies in response to infection and vaccination and lower levels in older men may impair ability to respond to pathogens. These differences in immune function between men and women may begin to further our understanding of how the immune system contributes to sex differences in healthspan and lifespan [18].

Although B cells were not significantly different between current smokers and non-smokers, many of the B cell subsets were. Specifically, the memory B cells (CD19+CD27+CD43-) were higher in smokers, while the percent of the memory B cells (and B cells in general) that express IgD+ and/or IgM+ were lower in smokers. Previous studies have shown similar results with memory B cells for current smokers and demonstrated that former smokers had memory B cells similar to that of never smokers. They additionally saw a similar class-switch in the memory B cells in current smokers, measured by a loss of IgM [35]. The switch from IgM to IgG, IgA or IgE is thought to be due to mature memory B cells responding to repeated antigen recognition [36], potentially due to neoantigens formed from smoke particles.

Equally important in this study is the lack of association of these immune cells with cardiovascular risk factors. While there were some weak associations with prevalent cardiovascular disease, diabetes, and hypertension, none remained significant using an FDR<0.05. These weak associations have been previously reported in studies with fewer immunophenotypes [13, 14, 20].

This study has several strengths. Properly stored PBMC samples for immune cell profiling came from participants in a richly characterized community-based cohort who were dementia-free. While fresh whole blood is the optimum media for immune cell profiling, data has shown that cryopreserved cells show excellent correlation to whole blood profiles [37]. Study participants have directly measured risk factors and *APOE* genotyping permitting association analyses that may provide a reference for future studies of immune cells and various clinical conditions. The participants in this study continue under surveillance for clinical outcomes and have up to 20 years of follow up data that can be used in future studies to examine the relationship between immune cell phenotypes and incident cardiovascular, cognitive, and other health outcomes. The study also has limitations. The FHS Offspring participants are predominantly white and European ancestry, and therefore do not reflect the diversity of the United States population. The immune cell profiling was conducted at one time point limiting our ability to examine longitudinal relations of changes in immune cell proportions with age. Circulating immune cells are not necessarily representative of tissue-resident T cells. Immune cell data was presented as a proportion rather than an absolute count, due to a lack of a complete blood cell count with differential, and this may explain differences in data compared with other reports. CMV was assayed at one point in time but was measured in mid to later adulthood when most exposures would have occurred [10]. Our study is cross-sectional and for some outcomes we may have limited power to detect modest associations with immune cells.

## CONCLUSIONS

We profiled 116 immune cell phenotypes including subtypes of CD4 and CD8 T cells, B cells, NK cells, and monocytes in the community-based Framingham Offspring cohort. Our observations confirm and extend known associations of immune cell subtypes with CMV and age that show a shift from a naïve phenotype towards an exhausted phenotype. We report sex differences, with males exhibiting a more exhausted, cytotoxic landscape than females. We identified associations between CD8 exhausted cells and B cell subsets, but not overall B cells, with smoking status. Importantly, we did not identify significant immune cell associations with other risk factors, such as body mass index, prevalent cardiovascular disease, hypertension or diabetes. While further studies in larger, more diverse sample and more than one time point with immunophenotypic data are needed, this work will provide a valuable resource for future studies of the association of immune cell phenotypes and incident age-related disease.

## MATERIALS AND METHODS

### Study sample

The FHS is a community-based prospective cohort study. Starting in 1948, the FHS recruited 5209 participants residing in Framingham, Massachusetts who were primarily white American adults of European ancestry as the Original cohort [38], followed by the Offspring cohort (n=5129) which was recruited in 1971, consisting of the children of the Original cohort and spouses of the Offspring [39, 40]. The Offspring cohort participants have been examined every 4-8 years since enrollment. A total of 3539 Offspring participants attended the seventh examination from 1998 to 2001, which is the first examination with existing stored PBMCs.

For the current study, we identified 1332 Offspring participants who attended exam 7 (1998 to 2001) and had 2 or more vials of PBMCs stored (to ensure the resource was not exhausted) that can be used to profile immune cells. Among this group of Offspring participants, we identified a study sample of 1000 individuals aged 40 years and older who were dementia-free at exam 7. Figure 2 is a flow chart showing participant inclusion and exclusion in the study. All participants provided written informed consent at each attended examination; FHS exams are reviewed and approved by the Institutional Review Board at Boston University Medical Center.

### Immunophenotyping methods

Immune cell phenotyping protocols followed previously published protocols used in other large population studies [41, 42]. Briefly, PBMCs were cryopreserved at the Framingham Heart Study Offspring Cohort Exam 7. The PBMCs were thawed in a 37° C water bath for 15 minutes. Cells were diluted to 10x initial volume by the slow addition of supplemented media with gentle mixing, pelleted, washed, and resuspended in phosphate-buffered saline and filtered. The filtered cells were divided into 5 assay panels for immunophenotyping. Fluorescent antibodies utilized for this study, manufacturer, catalog numbers, and matching isotypes used in specific assay panels are indicated in Supplementary Table 3.

For surface labeling panels (GDNK, MEMORY, MONOCYTES), samples are incubated with a fixable live/dead stain for 15 minutes. After centrifugation to remove the live/dead stain, cells were incubated with the indicated antibodies or appropriate isotype for 15 minutes in the dark at concentrations recommended by manufacturers. Cells were pelleted, washed with media, resuspended in 1% paraformaldehyde, and stored in the dark at 4C until run on the flow cytometer.

For intracellular staining, samples are stimulated with phorbal myristic acetate/Ionomycin in the presence of Brefeldin A for 3 hours at 37C as previously described [41, 42]. Cells are collected by centrifugation, washed, and incubated with CD3/CD4/CD8 antibodies for 15 minutes in the dark. Cells were washed and fixed with paraformaldehyde for 10 minutes. Cells were collected by centrifugation and incubated for 15 minutes with appropriate antibodies or isotype controls in the presence of saponin (a pore forming reagent). Cells were washed and stored in 2% paraformaldehyde in the dark at 4C until run on the flow cytometer.

Flow cytometry was performed on a MACSQuant 16. Single color compensation beads were used to set machine compensation. Isotype controls and FMO controls were used to aid in gate setting. FCS Express 6.0 was used to analyze data. A Vericell PBMC control sample (Biolegend) was analyzed with each run to assess run variability, with coefficients of variation (CVs) ranging from 2.5% for CD3+, 12.3% for CD4+, 17.5% for CD8+ and 7.8% for CD14+ monocytes.

All data are presented as a percent of their main parent population as listed in Supplementary Table 1. Major cell types analyzed for this study are displayed in Figure 1A–1E. and gating strategies utilized are shown in Supplementary Figures 3–7.

We excluded 13 immune cell phenotypes with very low variability (whose CV < 0.074 or IQR < 0.088, the 5% percentiles), leaving 116 cell phenotypes. The number of participants with data and summary statistics for each immune cell phenotype profiled are presented in Supplementary Table 2.

We calculated three ARIP measures using immune cell phenotypes profiled in our study: the CD4/CD8 ratio, and two measures of the ratio between naïve cells and memory and effector cells for both CD4 T cells and CD8 T cells. For the ratio between naïve T cells and memory T cells, Tn/Tm, we used the formula Tn/Tm = Tn / (Teff + Tem + Tcm) separately for the CD4 and CD8 Tn cells, Teff cells, Tem cells, and Tcm cells [17]. The number of participants with data and summary statistics of the three ARIP measures is summarized in Supplementary Table 2.

### Cytomegalovirus

CMV is a common pathogen that influences T cell subsets in individuals infected with CMV. Therefore, we assayed CMV IgG by ELISA using the Creative Diagnostics CMV IgG kit (Catalog # DEIA326R) using an existing plasma sample from Offspring exam 7 from 943 of the participants with immune cell phenotyping. We obtained quantitative values in U/ml; the CV for the CMV assay was 5.9%.

### Statistical analysis

Our analyses excluded 4 participants with missing data for most of the immune cell subtypes, resulting from failure of these samples for multiple assays. The 116 immune cell phenotypes had sample sizes ranging from n=922 to 996. The number of participants with data and summary statistics for each immune cell phenotype profiled are presented in Supplementary Table 2. Missingness in cell phenotypes data was due to poor sample quality, technical assay errors, or not enough cells to assay a specific subset. Number of missingness for each type is listed in Supplementary Table 2. We stratified age into five groups: age ≤ 55, 55 < age ≤ 60, 60 < age ≤ 65, 65 < age ≤ 70, and age > 70. We categorized CMV into 4 groups: negative (CM ≤ 11 U/ml), equivocal (11< CMV ≤ 15 U/ml), positive, below the limit of detection (LOD) (15 < CMV ≤ 800 U/ml) and positive, above the LOD (CMV > 800 U/ml). CMV was unavailable for 57 participants due to lack of available plasma sample from exam 7. Participant characteristics of individuals missing CMV values did not differ substantively from participants with CMV measured. For complete case analyses, individuals missing CMV status were omitted.

We computed unadjusted mean percentages of immune cell phenotypes for Table 1 immune cell types stratified by age, sex, and CMV group using complete-case data.

For all association analyses, immune cell phenotypes were rank-based inverse normal transformed and treated as the outcome variable. To test for association between CMV status and immune cell phenotypes, we used individuals with CMV status available and adjusted for age and sex (Model 1) using a linear mixed effect model, accounting for correlations among related individuals using a random effect with the kinship matrix.

To test for associations of immune cells with age, sex, smoking status as current smokers vs. non-smokers (never or former), and other covariates, we used a multivariable model (“Model 2”) that included CMV, age, sex, education level, *APOE* genotypes, and other CVD risk factors including BMI, smoking, presence of diabetes, prevalent CVD, prevalent AF, SBP, on hypertension treatment at exam 7, TC, HDL, on lipid treatment at exam 7, and prevalent cancer prior to exam 7. All covariates except *APOE* ε4 carrier status and education level were directly measured at the FHS Offspring exam 7. Education level was ascertained at FHS Offspring exams 2 and 8, and during off-exam-cycle neuropsychological testing. We used highest education level reported and categorized it into four levels: attended high school, high school graduate, attended college, and college graduate. *APOE* genotype groups were defined as ε3ε3, ε2 carriers, and ε4 carriers with the ε3ε3 group treated as the reference level. BMI was grouped into three levels: normal (BMI < 25), overweight (25 ≤ BMI < 30), and obese (BMI ≥ 30). The presence of diabetes was determined if any of the following were satisfied: fasting blood glucose level of 126 mg/dL or higher, random blood glucose level of 198 mg/dL or higher, or use of antidiabetic medications. Prevalent CVD was defined at exam 7 based on current or previous diagnosis of coronary heart disease (myocardial infarction, angina pectoris, coronary insufficiency), transient ischemic attack, intermittent claudication, and congestive heart failure determined by adjudication of a panel of senior investigators.

We used multiple imputation by chained equations [43] to create 10 imputed data sets to impute missing CMV values, immune cell phenotypes, and other missing data among the 996 participants in variables obtained from the Offspring exam 7, including the covariate data for the multivariable adjusted models (Model 2 covariates). In the imputation models, log-CMV levels were treated as quantitative with values above LOD set to the LOD. Age, sex, education level, *APOE* genotypes, all immune cell phenotypes, and available exam 7 variables were used in the imputation. For each immune cell phenotype outcome and each imputed data set, we fit a linear mixed effect model adjusting for Model 2 covariates accounting for correlations among related individuals using a random effect with the kinship matrix. Effect estimates and likelihood ratio p-values were pooled across data sets.

To account for multiple testing, false discovery rates [44] (FDRs) across the 116 immune cell phenotypes plus 3 ARIP measures were calculated separately for each predictor tested for association.

We used a threshold of FDR < 0.05 to declare significance. All analyses were conducted in R software version 4.0.2 [45]. The multiple imputations including pooling of the effect estimates and *p*–values from the fitted models were carried out using the mice package [43]. Linear mixed effect models were fitted using lmekin function from the coxme package [46].

## Supplementary Material

Supplementary Figures

Supplementary Table 1

Supplementary Table 2

Supplementary Table 3

Supplementary Table 4

Supplementary Table 5

Supplementary Table 6

Supplementary Table 7

Supplementary Table 8

Supplementary Table 9

Supplementary Table 10

Supplementary Table 11

Supplementary Table 12

Supplementary Table 13

Supplementary Table 14

Supplementary Table 15

Supplementary Table 16

Supplementary Table 17

Supplementary Table 18

## References

[1] López-Otín C, Blasco MA, Partridge L, Serrano M, Kroemer G. The hallmarks of aging. Cell. 2013; 153:1194–217. 10.1016/j.cell.2013.05.039 23746838 PMC3836174

[2] Zheng Y, Liu X, Le W, Xie L, Li H, Wen W, Wang S, Ma S, Huang Z, Ye J, Shi W, Ye Y, Liu Z, et al. A human circulating immune cell landscape in aging and COVID-19. Protein Cell. 2020; 11:740–70. 10.1007/s13238-020-00762-2 32780218 PMC7417788

[3] Márquez EJ, Trowbridge J, Kuchel GA, Banchereau J, Ucar D. The lethal sex gap: COVID-19. Immun Ageing. 2020; 17:13. 10.1186/s12979-020-00183-z 32457811 PMC7240166

[4] Pereira B, Xu XN, Akbar AN. Targeting Inflammation and Immunosenescence to Improve Vaccine Responses in the Elderly. Front Immunol. 2020; 11:583019. 10.3389/fimmu.2020.583019 33178213 PMC7592394

[5] Franceschi C, Capri M, Monti D, Giunta S, Olivieri F, Sevini F, Panourgia MP, Invidia L, Celani L, Scurti M, Cevenini E, Castellani GC, Salvioli S. Inflammaging and anti-inflammaging: a systemic perspective on aging and longevity emerged from studies in humans. Mech Ageing Dev. 2007; 128:92–105. 10.1016/j.mad.2006.11.016 17116321

[6] Goronzy JJ, Weyand CM. Successful and Maladaptive T Cell Aging. Immunity. 2017; 46:364–78. 10.1016/j.immuni.2017.03.010 28329703 PMC5433436

[7] Nikolich-Žugich J. The twilight of immunity: emerging concepts in aging of the immune system. Nat Immunol. 2018; 19:10–9. 10.1038/s41590-017-0006-x 29242543

[8] Hirokawa K, Utsuyama M, Hayashi Y, Kitagawa M, Makinodan T, Fulop T. Slower immune system aging in women versus men in the Japanese population. Immun Ageing. 2013; 10:19. 10.1186/1742-4933-10-19 23675689 PMC3663722

[9] Pawelec G, Derhovanessian E, Larbi A, Strindhall J, Wikby A. Cytomegalovirus and human immunosenescence. Rev Med Virol. 2009; 19:47–56. 10.1002/rmv.598 19035529

[10] Jergović M, Contreras NA, Nikolich-Žugich J. Impact of CMV upon immune aging: facts and fiction. Med Microbiol Immunol. 2019; 208:263–9. 10.1007/s00430-019-00605-w 31004198 PMC6635032

[11] Thyagarajan B, Faul J, Vivek S, Kim JK, Nikolich-Žugich J, Weir D, Crimmins EM. Age-Related Differences in T-Cell Subsets in a Nationally Representative Sample of People Older Than Age 55: Findings From the Health and Retirement Study. J Gerontol A Biol Sci Med Sci. 2022; 77:927–33. 10.1093/gerona/glab300 34633448 PMC9071411

[12] Patin E, Hasan M, Bergstedt J, Rouilly V, Libri V, Urrutia A, Alanio C, Scepanovic P, Hammer C, Jönsson F, Beitz B, Quach H, Lim YW, et al, and Milieu Intérieur Consortium. Natural variation in the parameters of innate immune cells is preferentially driven by genetic factors. Nat Immunol. 2018; 19:302–14. 10.1038/s41590-018-0049-7 29476184

[13] Olson NC, Doyle MF, Jenny NS, Huber SA, Psaty BM, Kronmal RA, Tracy RP. Decreased naive and increased memory CD4(+) T cells are associated with subclinical atherosclerosis: the multi-ethnic study of atherosclerosis. PLoS One. 2013; 8:e71498. 10.1371/journal.pone.0071498 24009662 PMC3751895

[14] Olson NC, Doyle MF, de Boer IH, Huber SA, Jenny NS, Kronmal RA, Psaty BM, Tracy RP. Associations of Circulating Lymphocyte Subpopulations with Type 2 Diabetes: Cross-Sectional Results from the Multi-Ethnic Study of Atherosclerosis (MESA). PLoS One. 2015; 10:e0139962. 10.1371/journal.pone.0139962 26458065 PMC4601795

[15] Adriaensen W, Pawelec G, Vaes B, Hamprecht K, Derhovanessian E, van Pottelbergh G, Degryse JM, Matheï C. CD4:8 Ratio Above 5 Is Associated With All-Cause Mortality in CMV-Seronegative Very Old Women: Results From the BELFRAIL Study. J Gerontol A Biol Sci Med Sci. 2017; 72:1155–62. 10.1093/gerona/glw215 27927759

[16] Wikby A, Månsson IA, Johansson B, Strindhall J, Nilsson SE. The immune risk profile is associated with age and gender: findings from three Swedish population studies of individuals 20-100 years of age. Biogerontology. 2008; 9:299–308. 10.1007/s10522-008-9138-6 18369735

[17] Ramasubramanian R, Meier HCS, Vivek S, Klopack E, Crimmins EM, Faul J, Nikolich-Žugich J, Thyagarajan B. Evaluation of T-cell aging-related immune phenotypes in the context of biological aging and multimorbidity in the Health and Retirement Study. Immun Ageing. 2022; 19:33. 10.1186/s12979-022-00290-z 35858901 PMC9297609

[18] Mittelbrunn M, Kroemer G. Hallmarks of T cell aging. Nat Immunol. 2021; 22:687–98. 10.1038/s41590-021-00927-z 33986548

[19] Wertheimer AM, Bennett MS, Park B, Uhrlaub JL, Martinez C, Pulko V, Currier NL, Nikolich-Žugich D, Kaye J, Nikolich-Žugich J. Aging and cytomegalovirus infection differentially and jointly affect distinct circulating T cell subsets in humans. J Immunol. 2014; 192:2143–55. 10.4049/jimmunol.1301721 24501199 PMC3989163

[20] Tracy RP, Doyle MF, Olson NC, Huber SA, Jenny NS, Sallam R, Psaty BM, Kronmal RA. T-helper type 1 bias in healthy people is associated with cytomegalovirus serology and atherosclerosis: the Multi-Ethnic Study of Atherosclerosis. J Am Heart Assoc. 2013; 2:e000117. 10.1161/JAHA.113.000117 23688675 PMC3698770

[21] Di Benedetto S, Derhovanessian E, Steinhagen-Thiessen E, Goldeck D, Müller L, Pawelec G. Impact of age, sex and CMV-infection on peripheral T cell phenotypes: results from the Berlin BASE-II Study. Biogerontology. 2015; 16:631–43. 10.1007/s10522-015-9563-2 25732234

[22] Vasunilashorn S, Ferrucci L, Crimmins EM, Bandinelli S, Guralnik JM, Patel KV. Association of inflammation with loss of ability to walk 400 meters: longitudinal findings from the Invecchiare in Chianti Study. J Am Geriatr Soc. 2013; 61:1743–9. 10.1111/jgs.12446 24083386 PMC3801413

[23] Goldeck D, Oettinger L, Janssen N, Demuth I, Steinhagen-Thiessen E, Pawelec G. Cytomegalovirus Infection Minimally Affects the Frequencies of B-Cell Phenotypes in Peripheral Blood of Younger and Older Adults. Gerontology. 2016; 62:323–9. 10.1159/000382076 26820888

[24] Puissant-Lubrano B, Apoil PA, Guedj K, Congy-Jolivet N, Roubinet F, Guyonnet S, Sourdet S, Nourhashemi F, Blancher A. Distinct effect of age, sex, and CMV seropositivity on dendritic cells and monocytes in human blood. Immunol Cell Biol. 2018; 96:114–20. 10.1111/imcb.1004 29359459

[25] Alpert A, Pickman Y, Leipold M, Rosenberg-Hasson Y, Ji X, Gaujoux R, Rabani H, Starosvetsky E, Kveler K, Schaffert S, Furman D, Caspi O, Rosenschein U, et al. A clinically meaningful metric of immune age derived from high-dimensional longitudinal monitoring. Nat Med. 2019; 25:487–95. 10.1038/s41591-019-0381-y 30842675 PMC6686855

[26] Xu B, Fan CY, Wang AL, Zou YL, Yu YH, He C, Xia WG, Zhang JX, Miao Q. Suppressed T cell-mediated immunity in patients with COVID-19: A clinical retrospective study in Wuhan, China. J Infect. 2020; 81:e51–60. 10.1016/j.jinf.2020.04.012 32315725 PMC7166040

[27] Qin C, Zhou L, Hu Z, Zhang S, Yang S, Tao Y, Xie C, Ma K, Shang K, Wang W, Tian DS. Dysregulation of Immune Response in Patients With Coronavirus 2019 (COVID-19) in Wuhan, China. Clin Infect Dis. 2020; 71:762–8. 10.1093/cid/ciaa248 32161940 PMC7108125

[28] Löhr P, Schiele S, Arndt TT, Grützner S, Claus R, Römmele C, Müller G, Schmid C, Dennehy KM, Rank A. Impact of age and gender on lymphocyte subset counts in patients with COVID-19. Cytometry A. 2023; 103:127–35. 10.1002/cyto.a.24470 34125495 PMC8426831

[29] Mogilenko DA, Shpynov O, Andhey PS, Arthur L, Swain A, Esaulova E, Brioschi S, Shchukina I, Kerndl M, Bambouskova M, Yao Z, Laha A, Zaitsev K, et al. Comprehensive Profiling of an Aging Immune System Reveals Clonal GZMK+ CD8+ T Cells as Conserved Hallmark of Inflammaging. Immunity. 2021; 54:99–115.e12. 10.1016/j.immuni.2020.11.005 33271118

[30] Focosi D, Bestagno M, Burrone O, Petrini M. CD57+ T lymphocytes and functional immune deficiency. J Leukoc Biol. 2010; 87:107–16. 10.1189/jlb.0809566 19880576

[31] Pachnio A, Ciaurriz M, Begum J, Lal N, Zuo J, Beggs A, Moss P. Cytomegalovirus Infection Leads to Development of High Frequencies of Cytotoxic Virus-Specific CD4+ T Cells Targeted to Vascular Endothelium. PLoS Pathog. 2016; 12:e1005832. 10.1371/journal.ppat.1005832 27606804 PMC5015996

[32] Zöphel D, Angenendt A, Kaschek L, Ravichandran K, Hof C, Janku S, Hoth M, Lis A. Faster cytotoxicity with age: Increased perforin and granzyme levels in cytotoxic CD8+ T cells boost cancer cell elimination. Aging Cell. 2022; 21:e13668. 10.1111/acel.13668 35818124 PMC9381916

[33] Márquez EJ, Chung CH, Marches R, Rossi RJ, Nehar-Belaid D, Eroglu A, Mellert DJ, Kuchel GA, Banchereau J, Ucar D. Sexual-dimorphism in human immune system aging. Nat Commun. 2020; 11:751. 10.1038/s41467-020-14396-9 32029736 PMC7005316

[34] Huang Z, Chen B, Liu X, Li H, Xie L, Gao Y, Duan R, Li Z, Zhang J, Zheng Y, Su W. Effects of sex and aging on the immune cell landscape as assessed by single-cell transcriptomic analysis. Proc Natl Acad Sci USA. 2021; 118:e2023216118. 10.1073/pnas.2023216118 34385315 PMC8379935

[35] Brandsma CA, Hylkema MN, Geerlings M, van Geffen WH, Postma DS, Timens W, Kerstjens HA. Increased levels of (class switched) memory B cells in peripheral blood of current smokers. Respir Res. 2009; 10:108. 10.1186/1465-9921-10-108 19909533 PMC2779187

[36] Edry E, Melamed D. Class switch recombination: a friend and a foe. Clin Immunol. 2007; 123:244–51. 10.1016/j.clim.2007.02.008 17500041

[37] Thyagarajan B, Barcelo H, Crimmins E, Weir D, Minnerath S, Vivek S, Faul J. Effect of delayed cell processing and cryopreservation on immunophenotyping in multicenter population studies. J Immunol Methods. 2018; 463:61–70. 10.1016/j.jim.2018.09.007 30222961 PMC6423980

[38] Dawber TR, Kannel WB. The Framingham study. An epidemiological approach to coronary heart disease. Circulation. 1966; 34:553–5. 10.1161/01.cir.34.4.553 5921755

[39] Feinleib M, Kannel WB, Garrison RJ, McNamara PM, Castelli WP. The Framingham Offspring Study. Design and preliminary data. Prev Med. 1975; 4:518–25. 10.1016/0091-7435(75)90037-7 1208363

[40] Kannel WB, Feinleib M, McNamara PM, Garrison RJ, Castelli WP. An investigation of coronary heart disease in families. The Framingham offspring study. Am J Epidemiol. 1979; 110:281–90. 10.1093/oxfordjournals.aje.a112813 474565

[41] Olson NC, Sitlani CM, Doyle MF, Huber SA, Landay AL, Tracy RP, Psaty BM, Delaney JA. Innate and adaptive immune cell subsets as risk factors for coronary heart disease in two population-based cohorts. Atherosclerosis. 2020; 300:47–53. 10.1016/j.atherosclerosis.2020.03.011 32209232 PMC7276206

[42] Bailin SS, McGinnis KA, McDonnell WJ, So-Armah K, Wellons M, Tracy RP, Doyle MF, Mallal S, Justice AC, Freiberg MS, Landay AL, Wanjalla C, Koethe JR. T Lymphocyte Subsets Associated With Prevalent Diabetes in Veterans With and Without Human Immunodeficiency Virus. J Infect Dis. 2020; 222:252–62. 10.1093/infdis/jiaa069 32052044 PMC7323499

[43] van Buuren S, Groothuis-Oudshoorn K. mice: Multivariate Imputation by Chained Equations in R. J Stat Softw. 2011; 45:1–67. 10.18637/jss.v045.i03

[44] Benjamini Y, Hochberg Y. Controlling the False Discovery Rate: A Practical and Powerful Approach to Multiple Testing. J R Stat Soc Ser B Methodol. 1995; 57:289–300. 10.1111/j.2517-6161.1995.tb02031.x

[45] R Core Team. R: A Language and Environment for Statistical Computing. 2020. https://www.r-project.org/

[46] Therneau TM. coxme: Mixed Effects Cox Models. 2020. https://CRAN.R-project.org/package=coxme

