# Correction for: Circulating immune cell phenotypes are associated with age, sex, CMV, and smoking status in the Framingham Heart Study offspring participants

**DOI:** 10.18632/aging.204988

**Published:** 2023-08-14

**Authors:** Yuan Fang, Margaret F. Doyle, Jiachen Chen, Jesse Mez, Claudia L. Satizabal, Michael L. Alosco, Wei Qiao Qiu, Kathryn L. Lunetta, Joanne M. Murabito

**Affiliations:** 1 Boston University School of Public Health, Department of Biostatistics, Boston, MA 02118, USA; 2 University of Vermont, Larner College of Medicine, Department of Pathology and Laboratory Medicine, Burlington, VT 05405, USA; 3 Boston University Chobanian and Avedisian School of Medicine, Boston University Alzheimer’s Disease Research Center and CTE Center, Boston, MA 02118, USA; 4 Boston University Chobanian and Avedisian School of Medicine, Department of Neurology, Boston, MA 02118, USA; 5 Framingham Heart Study, National Heart, Lung, and Blood Institute and Boston University Chobanian and Avedisian School of Medicine, Framingham, MA 01702, USA; 6 University of Texas Health Science Center at San Antonio, Glenn Biggs Institute for Alzheimer’s and Neurodegenerative Diseases, San Antonio, TX 78229, USA; 7 Boston University Chobanian and Avedisian School of Medicine, Department of Psychiatry, Boston, MA 02118, USA; 8 Boston University Chobanian and Avedisian School of Medicine, Department of Pharmacology and Experimental Therapeutics, Boston, MA 02118, USA; 9 Boston University Chobanian and Avedisian School of Medicine, Department of Medicine, Boston, MA 02118, USA; 10 Boston Medical Center, Department of Adult Primary Care, Boston, MA 02119, USA

**Keywords:** immune cell, CMV, aging, T cells, smoking

**This article has been corrected:** The authors found an error in **Table 2**, titled "Participant Characteristics." The table headers for the last two columns were mistakenly interchanged, with "Male" incorrectly labeled as "Female" and vice versa. The numbers and figures for males and females are correct in all the other tables. In addition, at one place in the text the authors refer to this table as follows: “Participant characteristics are summarized in Table 2. Among the 996 participants included in the study, 48% were female." This number corresponds to the number in the uncorrected Table 2 and should be corrected to read “52% were female.” This correction does not affect any of the conclusions in the manuscript.

The corrected **Table 2** and relevant text are presented below.

**Table 2:** Participant characteristics.

**Demographic**	**All**	**Female**	**Male**
**Sample size, n (%)**	996 (100%)	516 (52%)	480 (48%)
**Age, mean (range)**	62 (40, 88)	62 (41, 85)	62 (40, 88)
**CMV positive, n (%)**	465 (46.7%)	250 (48%)	215 (45%)
***APOE-ϵ4* carriers, n (%)**	222 (22.3%)	114 (22.1%)	108 (22.5%)
***APOE-ϵ2* carriers, n (%)**	140 (14.1%)	62 (12.0%)	78 (16.3%)
**Attended college, n (%)**	733 (73.6%)	363 (70.3%)	370 (77.1%)
**Current smoker, n (%)**	139 (14%)	89 (17%)	50 (10%)
**BMI kg/m2, mean (sd)**	28 (5)	28 (6)	28 (4)
**SBP mmHg, mean (sd)**	127 (18)	126 (20)	128 (17)
**DBP mmHg, mean (sd**	74 (10)	72 (10)	76 (10)
**Hypertension Rx, n (%)**	343 (34.4%)	156 (30.2%)	187 (39.0%)
**Total cholesterol mg/dL, mean (sd)**	199 (37)	206 (37)	192 (36)
**HDL mg/dL, mean (sd)**	53 (17)	60 (16)	46 (13)
**Lipid Rx, n (%)**	225 (22.6%)	96 (18.6%)	129 (26.9%)
**Fasting blood glucose mg/dL, mean (sd)**	105 (27)	101 (26)	108 (28)
**T2DM Rx, n (%)**	61 (6.1%)	28 (5.4%)	33 (6.9%)
**Prevalent CVD, n (%)**	142 (14.3%)	46 (8.9%)	96 (20.0%)
**Prevalent AF, n (%)**	42 (4.2%)	16 (3.1%)	26 (5.4%)
**Blood cancer prevalent exam 7, n (%)**	6 (1%)	5 (1%)	1 (0%)

Notes: CMV, cytomegalovirus; *APOE*, apolipoprotein E; BMI, body mass index; sd, standard deviation; SBP, systolic blood pressure; DBP, diastolic blood pressure; Rx, medication treatment; HDL, High-density lipoprotein cholesterol; CVD, cardiovascular disease; AF, atrial fibrillation; T2DM, type II diabetes.

## Participant demographics

Sample inclusion and exclusion criteria are shown in Figure 2. Participant characteristics are summarized in Table 2. Among the 996 participants included in the study, 52% were female.

