# Supplementary material for: Circulating immune cell phenotypes are associated with age, sex, CMV, and smoking status in the Framingham Heart Study offspring participants
Source: Aging (Albany NY). 2023 Apr 27;15(10):3939–66. doi: 10.18632/aging.204686 (PMC10258017; doi:10.18632/aging.204686)
Supplement: Supplementary Figures [file aging-15-204686-s001.pdf]

SUPPLEMENTARY FIGURES

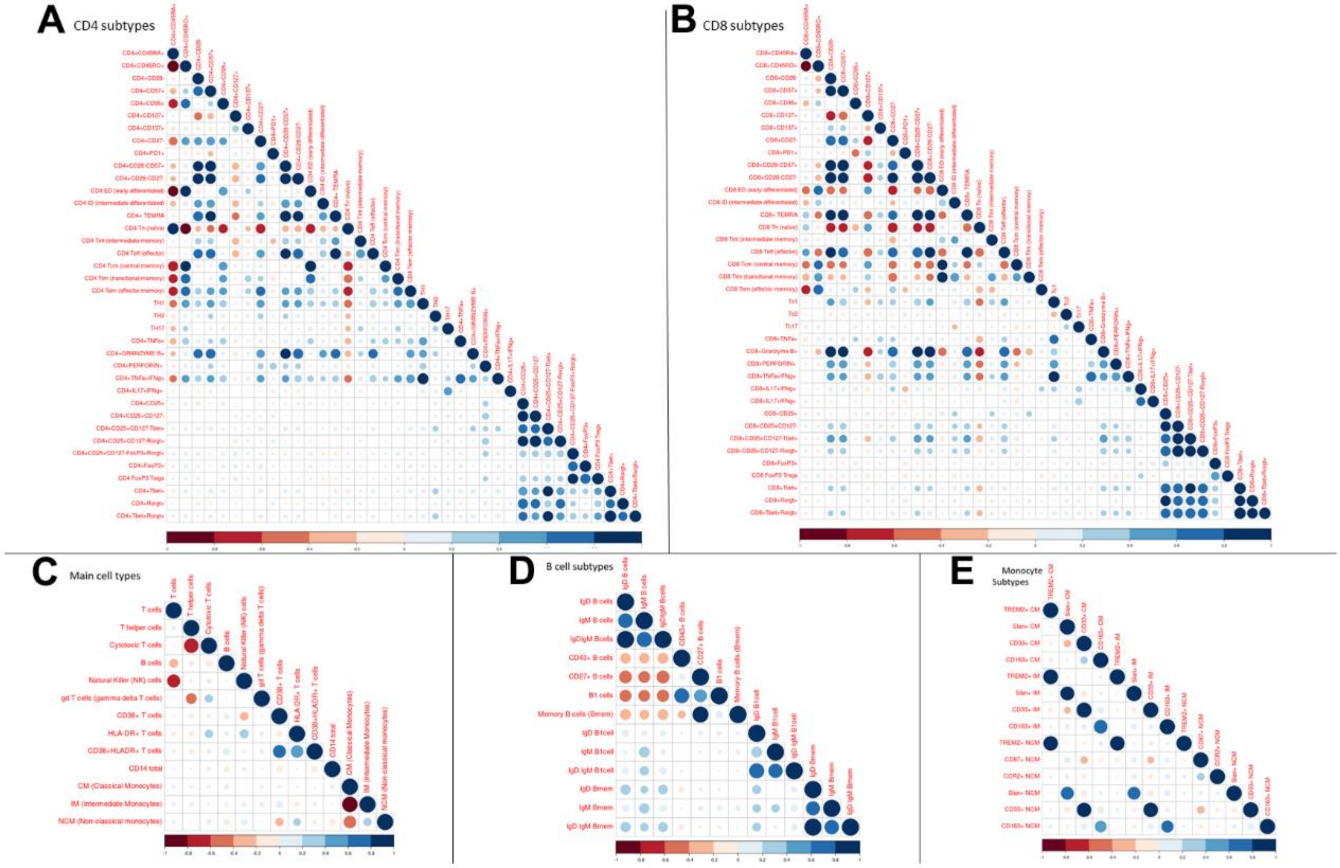

Supplementary Figure 1. Correlation plots of the immune cell phenotypes profiled grouped in subsets. (**A**) Spearman correlations among CD4 T helper cell subtypes. (**B**) Spearman correlations among CD8 cytotoxic T cell subtypes. (**C**) Spearman correlations between T cells, B cells, NK cells, monocytes, and their main subtypes profiled. (**D**) Spearman correlations among B cell subtypes. (**E**) Spearman correlations among subtypes of the classical monocytes, intermediate monocytes, and the non-classical monocytes.

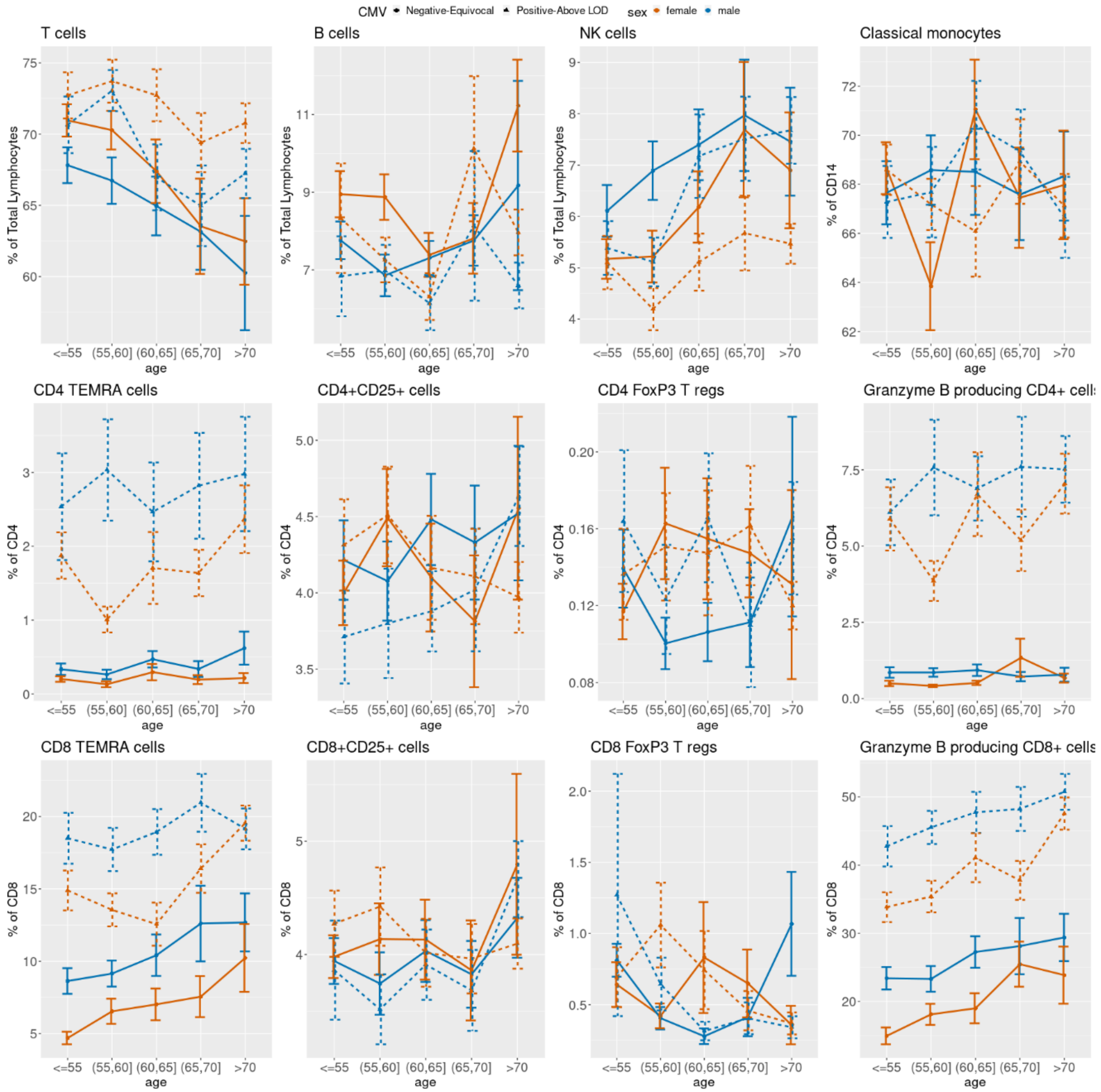

Supplementary Figure 2. Unadjusted mean percentages of CD3+ T cells, B cells, and NK cells of total live lymphocytes, unadjusted mean percentages of CD4 TEMRA cells, CD4 +CD25+cells, CD4 FoxP3 T reg cells, and Granzyme B producing CD4 cells, and unadjusted mean percentages of CD8 TEMRA cells, CD8+CD25+ cells, CD8 FoxP3 T reg cells, and Granzyme B producing CD8 cells across age groups, stratified by sex and two-group CMV status. Note: NK cells = Natural killer cells; TEMRA cells = CD45RA- re-expressing T effector memory cells; T regs cells = T regulatory cells.

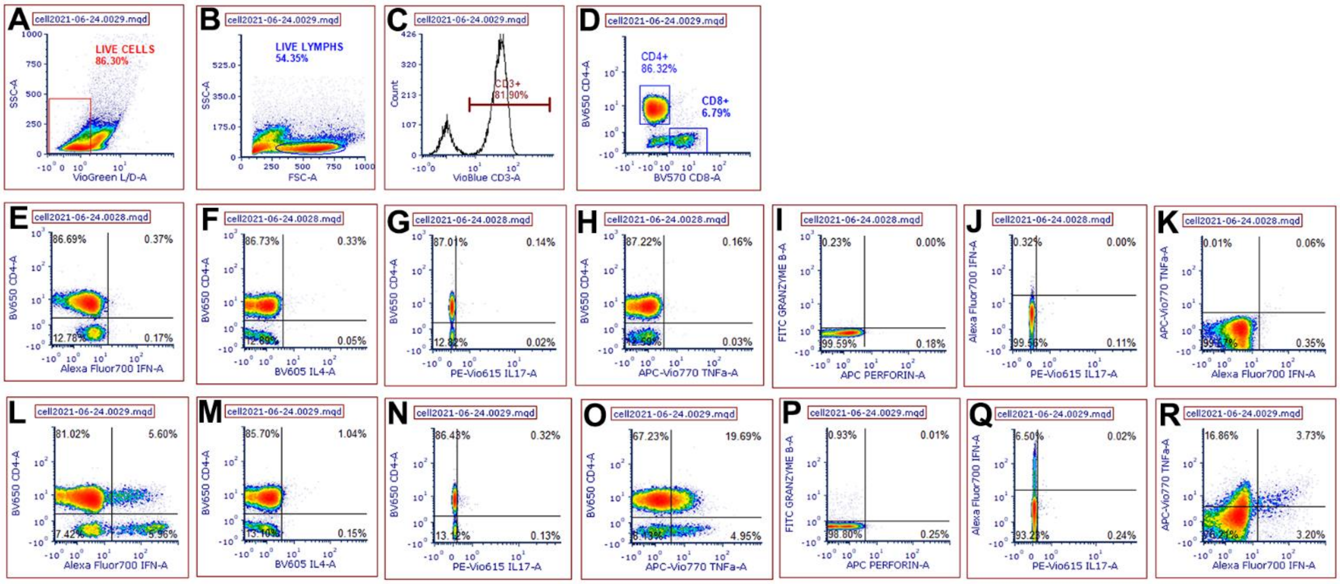

Supplementary Figure 3. Strategy for T cell stimulation gating. (**A**) gates out dead cells. (**B**) gates on only lymphocytes. (**C**) gates on CD3+ cells. (**D**) gates on CD4+ and CD8+ cells within the CD3+ cells. (**E**–**K**) are C3+CD4+ (or CD8+) with isotype controls used to set negative gates for IFNg, IL4, IL17, TNFa in the CD3+ gate, and Perforin/Granzyme B, IGNg/IL7, and TNFa/IFNg in the CD4+ (or CD8+) gate, respectively. (**L**–**R**) show full data. Data for CD8+ cells was gated identically.

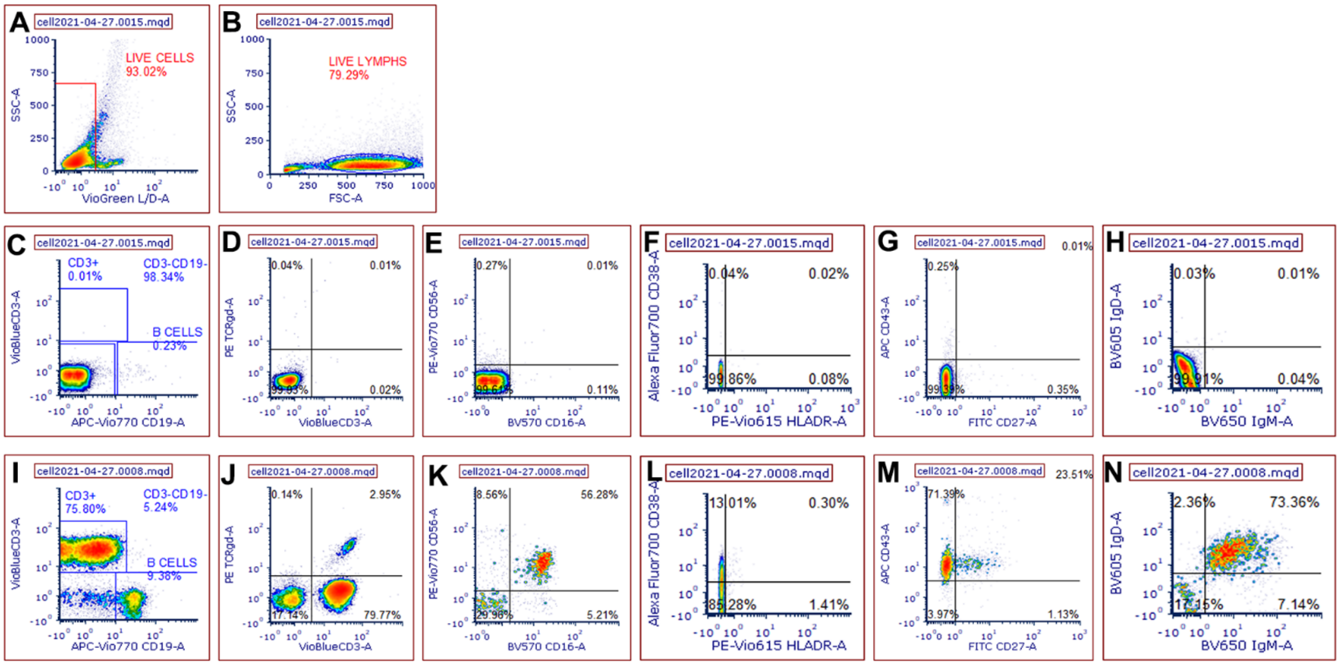

Supplementary Figure 4. Strategy for T, B, NK cell gating. PBMCs are gated on live cells (**A**), followed by lymphocytes (**B**). (**C**–**H**) are isotype controls. (**I**) is gated on live lymphocytes for CD3+, CD19+ and CD3-CD19-. (**J**) is gated on live lymphocytes for CD3+gdTCR+. (**K**) is gated on CD3-CD19- for CD56+CD16+ (NK cells). (**L**) is gated on CD3+ for CD38+ and HLADR+. (**M**) is gate on CD19+ for CD43+ and CD27+. (**N**) is gated on CD19+ for IgD and IgM.

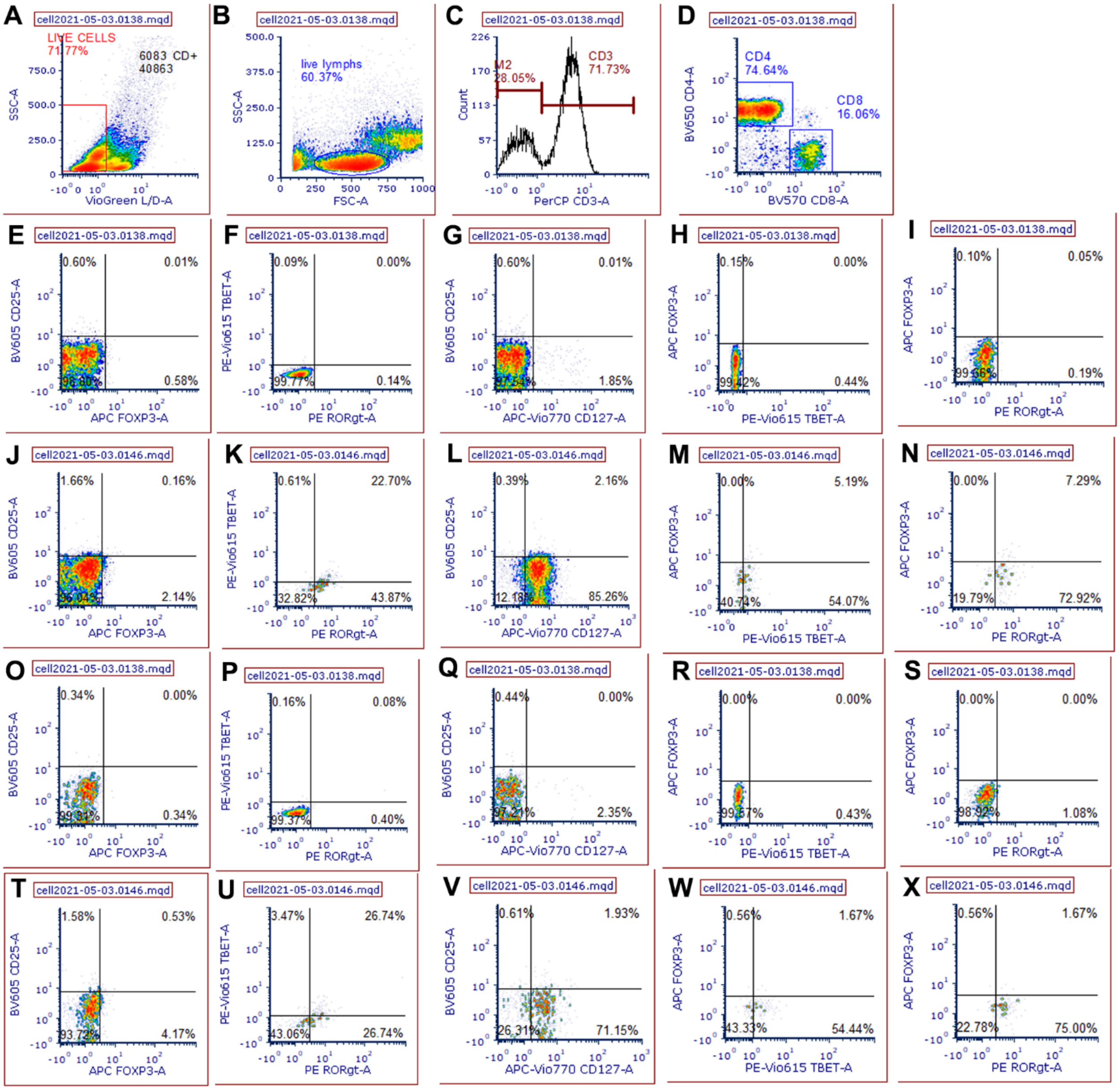

Supplementary Figure 5. Regulatory T cell gating strategy. PBMCs are gated on live cells (**A**), then lymphocytes (**B**), then CD3+ cells (**C**) and then on either CD4+ or CD8+ cells (**D**). Gating on CD4+ cells, isotype controls were used to set negative gates for CD25 and FoxP3 (**E**), tBET and RORgT (**F**), CD25 and CD127 (**G**), FoxP3 and tBET (**H**) and FoxP3 and RORgT (**I**). CD4+ cells were then analyzed for CD25 and FoxP3 (**J**), tBET and RORgT (**K**), CD25 and CD127 (**L**), FoxP3 and tBET (**M**) and FoxP3 and RORgT (**N**). Similar gating was used for CD8+ isotypes controls (**O**–**S**) and test samples (**T**–**X**).

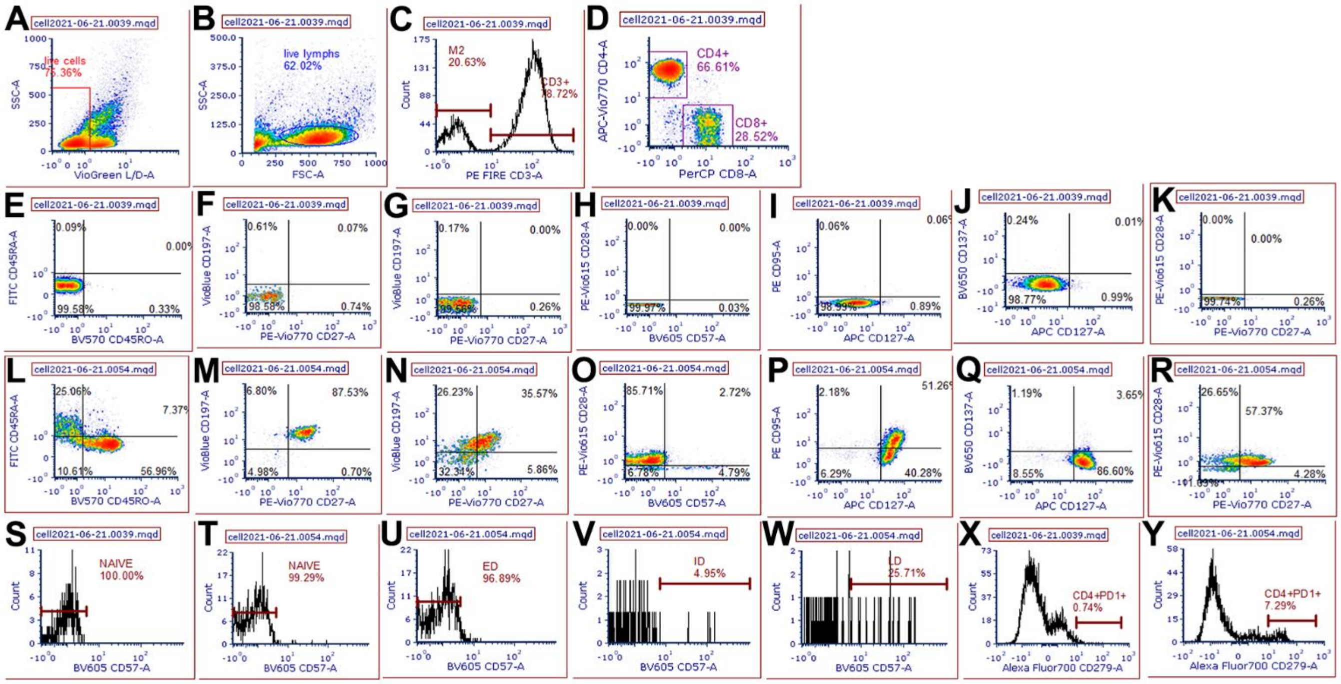

Supplementary Figure 6. Strategy for memory subset gating. PBMCs are gated on live cells (**A**), lymphocytes (**B**), CD3+ (**C**), CD4+ and CD8+ (**D**). Gating on CD4+, isotype controls were used to set negative gates for CD45RA and CD45RO (**E**), for CD45RA+: CD192 and CD27 (**F**), for CD45RO+: CD192 and CD27 (**G**), CD28 and CD57 (**H**), CD95 and CD127 (**I**), CD137 and CD127 (**J**), and CD28 and CD27 (**K**). CD45RA+ cells (upper left quadrant) (**L**) were used to gate CD197 and CD27 (**M**), while CD45RO+ (lower right quadrant) (**L**) were used to gate CD197 and CD27 (**N**). Gating on CD4+ cells, were positive samples for CD28 and CD57 (**O**), CD95 and CD127 (**P**), CD137 and CD127 (**Q**), and CD28 and CD27 (**R**). A CD57 isotype control (**S**) and CD57+ samples gated on CD4+CD45RA+CD28+CD27+ (**T**) for naïve cells, CD4+CD45RO+CD28+CD27+ (**U**) for early differentiated cells (ED), CD4+CD45RO+CD28+CD27+ (**V**) for intermediate differentiated cells (ID), and CD4+CD45RA+CD28-CD27- (**W**) for late differentiated cells (LD). Using the CD4+, is an isotype control (**X**) and a CD279+ (PD1) sample (**Y**). CD8+ memory subsets were performed using the same gating strategy.

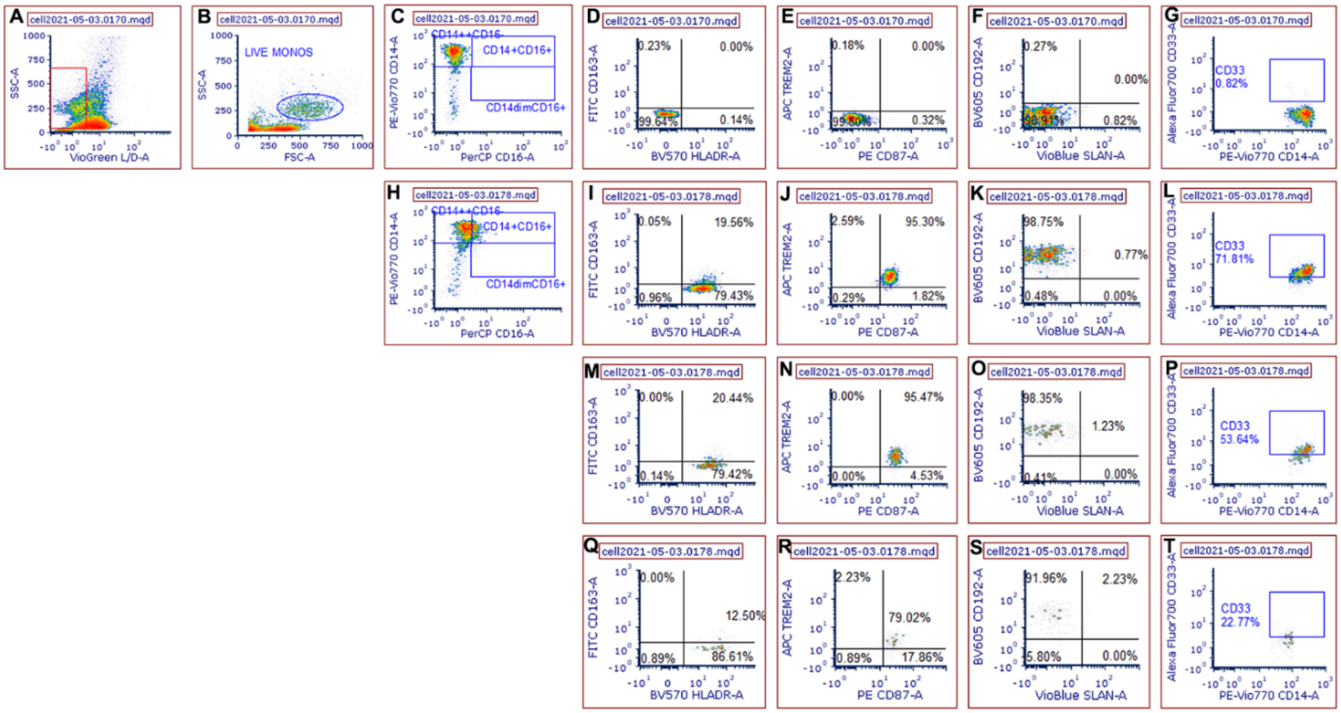

Supplementary Figure 7. Monocyte gating strategy. PBMCs are gated on live cells (**A**) followed by monocytes (**B**). (**C**) is a CD14+ sample used to set negative gates with isotypes. (**D**–**G**) are CD14+ cells with isotypes controls. (**H**) show gating for CD14+CD16- (Classical Monocytes), CD14+CD16+ (Intermediate Monocytes) and CD14dimCD16+ (non-Classical Monocytes). (**I**–**L**) are gated on Classical Monocytes for CD163 and HLADR (**I**), TREM2 and CD87 (**J**), CD192 and SLAN (**K**) and CD33 (**L**). (**M**–**P**) are the same as (**I**–**L**) except gated on intermediate monocytes. (**Q**–**T**) are the same as (**I**–**L**) except gated on the non-classical monocytes.
